# TurboFold II: RNA structural alignment and secondary structure prediction informed by multiple homologs

**DOI:** 10.1093/nar/gkx815

**Published:** 2017-09-28

**Authors:** Zhen Tan, Yinghan Fu, Gaurav Sharma, David H. Mathews

**Affiliations:** 1 Department of Biochemistry and Biophysics, University of Rochester Medical Center, 601 Elmwood Avenue, Box 712, Rochester, NY 14642, USA; 2 Center for RNA Biology, University of Rochester Medical Center, 601 Elmwood Avenue, Box 712, Rochester, NY 14642, USA; 3 Department of Electrical and Computer Engineering, University of Rochester, Hopeman 204, RC Box 270126, Rochester, NY 14627, USA; 4 Department of Biostatistics and Computational Biology, University of Rochester Medical Center, 601 Elmwood Avenue, Box 630, Rochester, NY 14642, USA

## Abstract

This paper presents TurboFold II, an extension of the TurboFold algorithm for predicting secondary structures for multiple RNA homologs. TurboFold II augments the structure prediction capabilities of TurboFold by additionally providing multiple sequence alignments. Probabilities for alignment of nucleotide positions between all pairs of input sequences are iteratively estimated in TurboFold II by incorporating information from both the sequence identity and secondary structures. A multiple sequence alignment is obtained from these probabilities by using a probabilistic consistency transformation and a hierarchically computed guide tree. To assess TurboFold II, its sequence alignment and structure predictions were compared with leading tools, including methods that focus on alignment alone and methods that provide both alignment and structure prediction. TurboFold II has comparable alignment accuracy with MAFFT and higher accuracy than other tools. TurboFold II also has comparable structure prediction accuracy as the original TurboFold algorithm, which is one of the most accurate methods. TurboFold II is part of the RNAstructure software package, which is freely available for download at http://rna.urmc.rochester.edu under a GPL license.

## INTRODUCTION

RNA is critical in cellular function. In addition to being the template for translation, RNA has been shown to be catalytic (1–3). Additionally, with increasing numbers of non-coding RNA (ncRNA) families being identified (4,5), there is strong interest in developing computational methods to estimate sequence alignment and secondary structure (6–12). These methods are key to detecting conserved regions (13–15), to understanding gene evolution (16) and to finding novel ncRNAs (17,18).

In protein alignment, homologous amino acids often conserve physical properties, such as polarity or hydrophobicity, even if the amino acid identity changes (19). Detecting homologous nucleotides in RNA is more difficult because of the simpler alphabet composition. A notable property of RNA alignments, however, is that they reflect the fact that secondary structure is conserved to a greater extent than sequence identity (20). Canonical base pairs between nucleotides are preserved by compensating mutations, for instance, from a GC pair to an AU pair or from a GC to a CG pair (21). Therefore, to increase accuracy, leading RNA alignment methods use secondary structure information (22–25).

There are several strategies for structural information-guided sequence alignment. One strategy is to solve the alignment and structure problems simultaneously, for example via dynamic programming using the Sankoff algorithm (26). The Sankoff algorithm is, however, computationally expensive, requiring *O*(*N*^3^*^H^*) time and *O*(*N*^2^*^H^*) memory, given *H* sequences with the average length *N*. A number of approaches have been used to accelerate these calculations, including restriction of the alignment (27,28) or structure space (29,30) or a simpler approximation to the problem using precomputed pair probabilities (22,31,32). Alternative structural alignment methods implement score function calculations based on sequence and structure similarity by comparison of upstream and downstream base pairing probabilities (33–35).

Another approach for improving multiple sequence alignments is to take the advantage of the homology across multiple sequences by using consistency among pairwise alignments (36,37). Probabilistic consistency, introduced by ProbCons (37), combines Hidden Markov Model (HMM)-based posterior probabilities with a heuristic that aims at three-way alignment consistency. The scoring of pairwise alignments is adjusted to favor the alignment of nucleotides to common nucleotides in the third sequence. In other words, given three homologous sequences, A, B and C, the alignment of A and C can be improved by having an alignment of A and B and also of B and C. Likewise, the other two pairwise alignments can be improved by such consistency. This can be extrapolated to consistency for a set of any number of sequences using three-way consistency of all sequence triples. ProbCons provides high alignment accuracy while maintaining fast computation speed (with complexity *O*(*H*^2^*N*^2^) in time, given *H* sequences with the average length *N*).

This paper describes TurboFold II, which is an extension of the original TurboFold algorithm (38). TurboFold predicts secondary structures for a set of homologous RNA sequences. Specifically, TurboFold iteratively estimates base pairing probabilities for each sequence using two types of information for sequence folding: *intrinsic information*, which is derived from the thermodynamic nearest neighbor model (39–41), and *extrinsic information*, which is inferred from other homologous sequences. The extrinsic information for a sequence is a proclivity for base pairing inferred from the posterior base pairing probabilities for other homologous sequences, mapped to the sequence of interest by the posterior probabilities of nucleotide co-incidence of the other sequences to that sequence. Two nucleotides are defined as *co-incident* when either they are aligned or when a nucleotide in one sequence occurs directly in a sequence of inserts following a nucleotide that aligns with a nucleotide in the other sequence (28). The posterior co-incidence probabilities are obtained with a Hidden Markov Model (HMM) for pairwise alignments (42). The estimated base pairing probabilities from TurboFold can be used to predict secondary structure for each sequence by three optional methods: thresholding the probabilities to compose a structure with base pairs with estimated base pairing probabilities higher than threshold, using the maximum expected accuracy (MEA) secondary structure prediction algorithm (43–45), or the ProbKnot method (46,47). TurboFold is iterative, with the extrinsic information being updated with each iteration, and the iterations were shown to improve the accuracy of the base pairing probability estimates. Because TurboFold does not strictly enforce the commonality among predicted structures, it also performs well on structurally diverged sequences.

TurboFold II makes several improvements upon the original TurboFold algorithm. Whereas TurboFold only provided secondary structure predictions, TurboFold II also provides a multiple sequence alignment that incorporates information from secondary structure conservation. In contrast with TurboFold that used fixed posterior coincidence probabilities computed at the start using only sequence information, TurboFold II updates the posterior co-incidence probabilities for inter-sequence alignment at each iteration. The updates incorporate secondary structure conservation information in the alignment by using a match score, calculated from estimated base pairing probabilities to represent the secondary structural similarity between nucleotide positions in the two sequences. Upon completion of the iterations, in addition to structure predictions computed as in TurboFold, TurboFold II computes a multiple sequence alignment that is progressively computed using a sum-of-pairs scoring based on a probabilistic consistency transformation, adopted from ProbCons (37).

To assess the performance of TurboFold II, the accuracy of sequence alignment and structure predictions were compared with several leading alignment tools, including pure sequence alignment methods, Clustal Omega (48); ClustalW (49); ProbCons (37), and also methods that do both alignment and structure prediction, LocARNA (22), MAFFT (50), MXSCARNA (23), and R-Coffee (51). In the comparison, TurboFold II shows significantly better alignment accuracy over other tools in the benchmark test for RNase P and telomerase RNA families. TurboFold II also outperforms several alignment methods except MAFFT on the SRP RNA family and except Clustal Omega and MAFFT on the small subunit ribosomal RNA (rRNA) family (where all tools are highly accurate). Furthermore, the structure prediction accuracy of TurboFold II is comparable to that of the original TurboFold algorithm.

## MATERIALS AND METHODS

### Base pairing probabilities and extrinsic information

TurboFold II uses an iterative framework analogous to TurboFold (38), taking homologous RNA sequences as input and providing estimates of base pairing probabilities for each sequence and alignment posterior probabilities for each pair of sequences as output (38). Prior to the iterations, pairwise posterior co-incidence probabilities and pairwise sequence identities are computed for each pair of sequences. Subsequent iterations compute updated estimates of: (a) base pairing probabilities using two sources of information: the nearest neighbor thermodynamic model of the sequence itself (called intrinsic information) and a combination of the estimated base pairing probabilities of other input sequence from previous iteration and the pairwise sequence alignment probabilities (called extrinsic information) and (b) posterior probabilities for alignment between nucleotide positions for each pair of sequences, again using two sources of information: the nucleotide identities for the sequence and a match score that quantifies the secondary structure similarity of nucleotide positions using the base pairing probabilities. For brevity, in the following description we drop the qualifier ‘estimated’ when referring to various probabilities.

**Figure 1.**
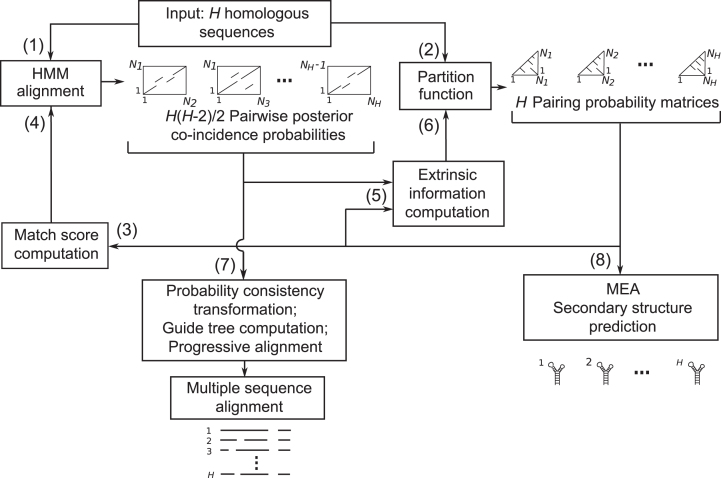
Flowchart for TurboFold II. The input is a set of homologous RNA sequences. In step 1, the pairwise posterior co-incidence probabilities (rectangular matrices) are calculated by pairwise HMM alignment. In step 2, base pairing probabilities (lower triangular matrices) are calculated using a partition function. In step 3, a match score is calculated for each sequence using the base pairing probabilities. In step 4, the coincidence probabilities are re-estimated using the match scores. In step 5, the base pairing probabilities and coincidence probabilities are used to calculate extrinsic information for each sequence, and, in step 6, the base pairing probabilities are re-estimated using the extrinsic information. Steps 3, 4, 5 and 6 form a loop that is used for multiple iterations. At step 7, a probabilistic consistency transformation is used to estimate a multiple sequence alignment. And at step 8, structures are estimated for each sequence.

As illustrated in Figure 1, TurboFold II comprises eight main steps: (1) computing pairwise posterior co-incidence probabilities using an HMM, (2) estimating base pairing probabilities using a partition function, (3) calculating an alignment match score (ρ) for each possible pair of nucleotide positions for each pair of sequences, (4) re-computing posterior co-incidence probability matrices that incorporate the match score, (5) calculating extrinsic information for each sequence by combining base pairing probabilities from other input sequences using the posterior co-incidence probabilities, (6) re-computing estimated base pairing probabilities by a partition function, using extrinsic information by combining updated posterior co-incidence probabilities and base pairing probabilities, (7) re-estimating the pairwise comparison score by probabilistic consistency transformation, building a guide tree, and performing progressive alignment and (8) predicting final secondary structures. Steps (3), (4), (5) and (6) form a loop that is iterated through multiple times. Each step is described below in more detail. The *H* homologous sequences are denoted by ${X_1}$, ${X_2}$,… ${X_H}$ with corresponding lengths ${N_1}$, ${N_2}$,… ${N_H}$, respectively.

#### Initial posterior co-incidence probability

Pairwise posterior co-incidence probabilities are estimated for all pairs of sequences with an HMM as implemented by Harmanci *et al.* (28). In the HMM, an alignment between two sequences is specified by a sequence of three states: aligned nucleotide positions (ALN); an insertion in the first sequence (INS1), a nucleotide in first sequence but no corresponding nucleotide in the second sequence; and an insertion in the second sequence (INS2). HMM parameters are the state transition probabilities for these three states that represent the pairwise alignment and the emission probabilities for the nucleotides in the sequences. Using the forward-backward algorithm, matrices of posterior co-incidence probabilities for two nucleotides (one from each sequence) are calculated. Detailed descriptions of co-incidence, posterior probabilities for pairwise alignment, and HMM parameter optimization can be found in (28).

#### Base pairing probabilities

Base pairing probabilities are calculated using the partition function method in RNAstructure (52).

#### Match score (ρ)

TurboFold II improves upon TurboFold by updating the pairwise posterior co-incidence probabilities during the iterations instead of using a static set of pre-computed probabilities. To provide sequence alignments that conform better with predicted secondary structures, the pairwise posterior co-incidence probabilities are recomputed during each iteration while incorporating a prior probability for base pairings based on a match score that encourages alignment between nucleotide positions where both nucleotides are either upstream paired, downstream paired, or unpaired. A nucleotide position in a sequence is said to be upstream or downstream paired, respectively, if it is paired with another nucleotide that is closer to the 5′ or 3′ end of the sequence. The details of the match score follow.

A match score for alignment based on base pairing probabilities was proposed in PMcomp (35), and this is adapted and utilized here as a prior. For the *m*th sequence, based on estimated base pairing probabilities between all pairs of nucleotide positions obtained from the partition function calculation, for a nucleotide at position $i$, the estimated probability of downstream pairing is $P_ < ^m\ ( i ) = \mathop \sum \nolimits_{j >i} P_{ij}^m$, of upstream pairing is $P_ >^m\ ( i ) = \mathop \sum \nolimits_{j < i} P_{ij}^m$, and of being unpaired is $P_{\circ} ^m\ ( i ) = {\rm{\ }}1 - P_ < ^m( i ) - P_ >^m( i )$. In alignments between two homologous sequences with conserved secondary structures, aligned nucleotide positions typically have the same status: both aligned nucleotides are upstream paired, downstream paired, or unpaired. Therefore, to encourage alignments that conform better with estimated base pairing probabilities for secondary structures, an alignment match score between nucleotides *i* and *k* in sequences m and n, respectively, is formulated as

(1) \begin{eqnarray*} &&{\rm{\rho }}\left( {i,k} \right) \nonumber \\ &&= {\alpha _1}\ \left( {\sqrt {P_ < ^m\left( i \right)P_ < ^n\left( k \right)} + \sqrt {P_>^m\left( i \right)P_ > ^n\left( k \right)} } \right) + {\alpha _2}\left( {\sqrt {P_{\circ}^m\left( i \right)P_{\circ}^n\left( k \right)} } \right) + {\alpha _3} \end{eqnarray*}

where ${\alpha _1}$ and ${\alpha _2}$ are nonnegative weight parameters that determine the emphasis placed on requiring that paired and unpaired nucleotides are aligned, respectively, and ${{\boldsymbol{\alpha }}_3}$ is the nonnegative parameter that controls the ratio of match scores between the situation where a paired nucleotide aligns with an unpaired nucleotide and the situation where two paired or unpaired nucleotides align. Both of these situations are encountered near the boundary of stems and loops in RNA structures, and the introduction of ${\alpha _3}$ can therefore improve the overall alignment accuracy. This computation step scales *O*(*H*^2^*N*^2^) in time, where *H* is the number of sequences and *N* is the length of each sequence.

Maximization of the alignment match score in Equation (1) encourages alignments that conform better with predicted base pairing probabilities for secondary structure and therefore can be used to inform alignment based on secondary structures. This was first proposed in PMcomp (35), which used a specific instance of the match score of Equation (1) obtained by setting ${\alpha _1} = {\alpha _2} = {\boldsymbol{\ }}1$ and ${\alpha _3} = {\boldsymbol{\ }}0$. Whereas PMcomp utilized the match score directly in a dynamic programming-based maximization, here we incorporate the match score as a prior in the HMM based computation of posterior probabilities for alignment between nucleotide positions, which are then iteratively updated.

#### Updating posterior co-incidence probabilities

In step 4, information from prior iterations is utilized to re-estimate alignment posterior probabilities and base pairing probabilities for secondary structures. The iterative re-estimation of alignment posterior probabilities is new to TurboFold II and uses the standard HMM alignment model (42), but with the match score of Equation (1) incorporated as a prior. This is complementary, yet analogous, to the incorporation of extrinsic information, in TurboFold, as a prior for the partition function based re-estimation of base pairing probabilities. The framework for HMM based pairwise alignment of homologous sequences is already extensively covered in (42). The description here highlights the new elements in TurboFold II following the notational conventions from Harmanci *et al.* (28).

The pairwise alignment HMM modeling the two homologous RNA sequences ${X_m}$ and ${X_n}$ progresses through a series of stochastic state transitions between states in the set $\{$ALN, INS1, INS2} corresponding to alignment, insertion in sequence 1, and insertion in sequence 2, respectively. Nucleotides observed in the sequences arise from HMM emissions where in the ALN state, a nucleotide is emitted for each sequence and in the insertion states, a nucleotide is emitted for the sequence with the insertion and an unobserved gap symbol ‘-’ for the other sequence. The HMM enables efficient computation of the posterior co-incidence probability $P( {i\sim k{\rm{|}}{X_m},{X_n}} )$ that nucleotide $i$ in sequence ${X_m}$ is co-incident with nucleotide $k$ in sequence ${X_n}$ via the recursive computation of the so-called forward and backward variables. The forward-variable ${\alpha _{{S_t}}}( {i,k} )$ is the probability the HMM produces the first $i$ and $k$ nucleotides, respectively, from the first and second sequence and is in state ${S_t}$, where ${S_t}$$ \in \{$ALN, INS1, INS2}. The backward variable ${\beta _{{S_t}}}( {i,k} )$ is the probability that conditioned on starting in the state ${S_t}$ the HMM produces the nucleotides $i$+*1* through ${N_m}$ and $k$+*1* through ${N_n}$, respectively, from the first and second sequence.

TurboFold II computes the forward variable using the recursions

(2) \begin{equation*}\begin{array}{@{}*{1}{l}@{}} {{\alpha _{ALN}}(i,k) = \sum\limits_{{S_t} \in \left\{ {ALN,INS1,INS2} \right\}} {{\rm{\tau }}\left( {{S_t},ALN} \right){{\rm{\gamma }}_{ALN}}\left( {X_1^i,X_2^k} \right)\rho \left( {i,k} \right){\alpha _{{S_t}}}\left( {i - 1,k - 1} \right)} }\\ {{\alpha _{INS1}}(i,k) = \sum\limits_{{S_t} \in \left\{ {ALN,INS1,INS2} \right\}} {{\rm{\tau }}\left( {{S_t},INS1} \right){{\rm{\gamma }}_{INS1}}\left( {i, - } \right){\alpha _{{S_t}}}\left( {i - 1,k} \right)} }\\ {{\alpha _{INS2}}\ \left( {i,k} \right) = \sum\limits_{{S_t} \in \left\{ {ALN,INS1,INS2} \right\}} {{\rm{\tau }}\left( {{S_t},INS2} \right){{\rm{\gamma }}_{INS2}}\left( { - ,k} \right){\alpha _{{S_t}}}\left( {i,k - 1} \right)} } \end{array}\end{equation*}

where ${\rm{\tau }}( {{S_{t + 1}},{S_t}} )$ denotes the conditional probability that the next state is ${S_{t + 1}}$ given the current state is ${S_t}$, ${{\rm{\gamma }}_{{S_t}}}( {{\rm{Y}},Z} )$ for Y, Z $ \in \{$A, C, G, U,-} is probability for emission of the pair ${\rm{Y}},Z$ in the state ${S_t}$, and, as described earlier, $\rho ( {i,k} )$ is the match score for secondary structure similarity between nucleotide positions $i$ and $k$, which incorporates the estimated structural information into the HMM alignment process. The backward variable recursions in TurboFold II are given by

(3) \begin{equation*}\begin{array}{@{}*{1}{l}@{}} {{\beta _{{S_t}}}\ \left( {i,k} \right) = \ {\rm{\tau }}\left( {{S_t},ALN} \right){{\rm{\gamma }}_{ALN}}\left( {i,k} \right)\rho \left( {i,k} \right){\beta _{ALN}}\left( {i + 1,k + 1} \right)}\\ { + {\rm{\tau }}\left( {{S_t},INS1} \right){{\rm{\gamma }}_{INS1}}\left( {i, - } \right){\beta _{INS1}}\left( {i + 1,k} \right)}\\ { + {\rm{\tau }}\left( {{S_t},INS2} \right){{\rm{\gamma }}_{INS2}}\left( { - ,k} \right){\beta _{INS1}}\left( {i,k + 1} \right)} \end{array}\end{equation*}

Compared with TurboFold the new component in Equations (2) and (3) is the introduction of the match score, $\rho ( {i,k} )$. In the HMM framework, the match scores $\rho ( {i,k} )$in Equations (2) and (3) correspond (after normalization) to a prior probability for pairing of nucleotide positions $i$ in sequence with nucleotide positions and $k$. Incorporation of the score, $\rho ( {i,k} )$, increases the likelihood of alignment of nucleotide positions $i$ and $k$ if both positions have higher probability of being in the same structural pairing state (both upstream-paired, downstream-paired, or unpaired) compared with the case when the structural pairing states of positions $i$ and $k$ are different.

Once the forward and backward variables have been recursively computed, the posterior co-incidence probability can be obtained from these as (28)

(4) \begin{equation*}P\ \left( {i\sim k{\rm{|}}{X_m},{X_n}} \right) = \frac{{\mathop \sum \nolimits_{{S_t} \in \left\{ {ALN,INS1,INS2} \right\}} {\alpha _{{S_t}}}\left( {i,k} \right){\beta _{{S_t}}}\left( {i,k} \right)}}{{\mathop \sum \nolimits_{{S_t} \in \left\{ {ALN,INS1,INS2} \right\}} {\alpha _{{S_t}}}\left( {{N_m},{N_n}} \right)}}\ \end{equation*}

Alignment posterior probabilities required for the probabilistic consistency transformation in Step (7) are also obtained from the forward and backward variables as

(5) \begin{equation*}P{\rm{\ }}\left( {i - k{\rm{|}}{X_m},{X_n}} \right) = \frac{{{\alpha _{ALN}}\left( {i,k} \right){\beta _{ALN}}\left( {i,k} \right)}}{{\mathop \sum \nolimits_{{S_t} \in \left\{ {ALN,INS1,INS2} \right\}} {\alpha _{{S_t}}}\left( {{N_m},{N_n}} \right)}}{\rm{\ }}\end{equation*}

#### Extrinsic information

The extrinsic information calculation begins with computing base pairing proclivity for each sequence, inferred from every other sequence. For each sequence, a lower triangular matrix is calculated. Specifically, the proclivity ${P^{( {n \to m} )}}( {i,\ j} )$ for base pairing between nucleotide positions $i$ and $j$ in sequence $m$ inferred from sequence *n* is computed as

(6) \begin{equation*}{P^{\left( {n \to m} \right)}}\ \left( {i,\ j} \right) = \mathop \sum \limits_{\begin{array}{@{}*{1}{c}@{}} {k,l}\\ {1 \le k < l \le {N_n}}\\ {k \in C_i^{m,n}}\\ {l \in C_j^{m,n}} \end{array}} {P^n}\left( {k,l} \right) \times {P^{\left( {m,n} \right)}}\left( {i\sim k} \right) \times {P^{\left( {m,n} \right)}}\left( {j\sim l} \right)\ \end{equation*}

where ${P^n}( {k,\ l} )$ is the probability of pairing between nucleotide positions $k$ and $l$ in sequence *n*, ‘$i\sim k$’ indicates the alignment between the nucleotides at indices$\ i$ and $k$ in the two sequences with ${P^{( {m,n} )}}( {i\sim k} )$ denoting the corresponding probability, and $C_i^{m,n}$ and $C_j^{m,n}$ denote the sets of indices outside of which the posterior co-incidence alignment probabilities ${P^{( {m,n} )}}( {i\sim k} )$ and ${P^{( {m,n} )}}( {j\sim l} )$, respectively, are smaller than 0.01. Exclusion of indices outside of the sets $C_i^{m,n}$ and $C_j^{m,n}$ from the summation in Equation (6) saves computation time without a significant accuracy performance penalty.

The extrinsic information ${\tilde{P}^m}$ for sequence *m* is then obtained as the normalized sum of the proclivities for the sequence *m* inferred from all other sequences, where the proclivities are inversely weighted by the pairwise sequence identity. That is,

(7) \begin{equation*}{\tilde{P}^m} = {\alpha ^m}\ \mathop \sum \limits_{n \in N\backslash m} \left( {1 - {\psi _{m,n}}} \right) \times {P^{\left( {n \to m} \right)}}\end{equation*}

where ${\psi _{m,n}}$ is the identity between sequences $m$ and $n$ computed from the HMM alignment, and ${\alpha ^m}$ is a normalizing factor that sets the maximum value in ${\tilde{P}^m}$ as one. The extrinsic information for each sequence is then normalized by the maximum pair extrinsic information for that sequence. A detailed description is in Harmanci *et al.* (38).

#### Updating extrinsic information and base pairing probabilities

The extrinsic information (the normalized sum of the base pairing proclivities for all pairs of each sequence with other sequences) is re-computed as in step (5), using updated posterior co-incidence probabilities (from step 4) and base pairing probabilities (from step 2).

Repeating step (2), the partition function is re-computed with the extrinsic information. The extrinsic information is incorporated as a pseudo free energy term in the partition function calculation. A detailed description is in Harmanci *et al.* (38).

#### Probabilistic consistency transformation, guide tree computation, progressive alignment, and computing final multiple sequence alignment

Upon completion of the iterations, using the posterior co-incidence probabilities obtained with the most recent match scores through step (3) are used to obtain a multiple sequence alignment.

Probabilistic consistency, which was described in ProbCons (37), is based on three-way alignment consistency of pairwise HMM posterior probabilities. From the pairwise HMM alignments, for each pairwise alignment, between sequences ${X_m}$ and ${X_n}$, the alignment score between two nucleotides $i$ and $k$ (the *i*th nucleotide of sequence ${X_m}$, and *k*th nucleotide of sequence ${X_n}$) are calculated based on probabilistic consistency transformation

(8) \begin{equation*}{\boldsymbol{P^\prime\ }}\left( {i\sim k{\rm{|}}{X_m},{X_n}} \right) = \frac{1}{{\left| S \right|}}\ \mathop \sum \limits_{{X_o} \in S} \mathop \sum \limits_q {\boldsymbol{P}}\left( {i\sim q{\rm{|}}{X_m},{X_o}} \right){\boldsymbol{P}}\left( {q\sim k{\rm{|}}{X_o},{X_n}} \right)\end{equation*}

where ${\boldsymbol{P^\prime}}( {i\sim k \in {{\boldsymbol{a}}^*}{\rm{|}}{X_m},{X_n}} )$ is the re-estimated alignment score of sequences ${X_m}$and ${X_n}$, $q$ is the $q$th nucleotide in sequence ${X_o}$. Re-estimated alignment scores are used in progressive alignments, which are processed hierarchically according to a guide tree as described in ProbCons (37).

#### Structure prediction using updated base pair probabilities

The structures are predicted by the maximum expected accuracy algorithm. Given the base pair probabilities ${P^m}( {i,\ j} )$ for sequence ${X_m}$, the maximum expected accuracy structure is defined as

(9) \begin{equation*}S_m^* = \mathop {{\rm{argmax}}}\limits_{{S_m}} \left\{ {\mathop \sum \limits_{(i,j) \in {S_m}} 2 \cdot {P^m}(i,j) + \mathop \sum \limits_{\begin{array}{@{}*{1}{c}@{}} {\forall i;}\\ {i\;{\rm unpaired}\;{\rm in}\;{S_m}} \end{array}} {P^m}(i)} \right\}\end{equation*}

where ${P^m}( i )$ is the probability that nucleotide position $i$ is not base paired, which is computed as

(10) \begin{equation*}{P^m}\ \left( i \right) = \ 1 - \mathop \sum \limits_{j\ = \ i + 1}^{{N_m}} {P^m}\left( {i,j} \right) - \mathop \sum \limits_{j\ = \ 1}^{i - 1} {P^m}\left( {j,i} \right)\end{equation*}

The MEA structure is obtained with a dynamic programming algorithm as described in (38).

### Parameter optimization

For parameter optimization and benchmarking, an RNA alignment and structure database, named RNAStralign (http://rna.urmc.rochester.edu), was aggregated from available online databases of RNA structure and alignment. Compared with the pre-existing BRAliBase dataset (53), RNAStralign has greater diversity of sequences; in particular, several sequence families longer than 320 nucleotides are included.

Structures for each family in RNAStralign are categorized into homologous families based on the classifications in the original databases. If available, further categorization into subfamilies was also included in RNAStralign. Only sequences with known alignments and secondary structures were included. The families included are 5S ribosomal RNA (54), Group I intron (55), tmRNA (56), tRNA (57), 16S ribosomal RNA (58), Signal Recognition Particle (SRP) RNA (59), RNase P RNA (60) and telomerase RNA (61).

To train the three parameters in the match score scheme (${\alpha _1}$, ${\alpha _2}$, and ${\alpha _3}$), 40 groups of input sequences, comprising three, five and seven homolog sets, were randomly chosen from RNAStralign for the 5S ribosomal RNA (Eubacteria subfamily), group I intron (IC1 subfamily), tmRNA, and tRNA families. A search was performed to find optimal parameter values for these selected sequences over a 3D grid with ${\alpha _1}$ and ${\alpha _2}$ for values 0, 0.6, 0.8, 1.0, 2.0, 3.0, 4.0, and 5.0, and ${\alpha _3}$ for values 0, 0.3, 0.5, 0.7 and 1.0. The resulting optimal parameters (${\alpha _1}$ = 1.0, ${\alpha _2}$ = 0.8, ${\alpha _3}$ = 0.5) were then used as the defaults for the TurboFold II. Supplementary Figure S2 illustrates the landscape for the grid search. The HMM parameters and the alignment constraint thresholds (the cutoff value below which co-incidence probabilities were excluded from the extrinsic information sum in order to reduce computational time) were kept identical to those used for TurboFold (38).

### Benchmarks

Default options and parameters were used for the other programs use in the benchmarking. For RNAalifold (2.1.9), separate benchmarks were run using Clustal Omega (1.2.1), (48) or ClustalW (2.1) (49) to predict input alignments (62).

For benchmarking, groups of sequence homologs were randomly selected from families distinct from those used for estimation of the parameters. Specifically, 200 groups of 5, 10 or 20 sequence homologs were selected from the small subunit ribosomal RNA (Alphaproteobacteria subfamily), SRP RNA (Protozoan subfamily), RNase P RNA (bacterial type A subfamily) and telomerase RNA. For SRP RNA, sequences shorter than 200 nucleotides were excluded because their structures are not consistent with those of longer sequences. All methods were benchmarked on the same groups of sequences, except for the single-sequence predictions, which were obtained by running MaxExpect from RNAstructure 5.7 (45,63) on each available sequence.

To allow for comparison against previous evaluations, benchmarks for the commonly used BRAliBase dataset (53), which provides multiple sequence alignments categorized by sequence identity, are included in the Supplementary Materials (Supplementary Figure S3). BRAliBase suffers from a bias in the ‘twilight zone’ sequence identities ranging from 40% to 60%, caused by the fact that a majority of sequences in BRAliBase for this range of sequence identities are tRNAs (64). Therefore, alignment methods with a performance advantage for tRNA demonstrate better performance in the low similarity region for BRAliBase.

### Comparison with other methods that align sequences with structure as auxiliary information

Like TurboFold II, the MAFFT (50) and R-Coffee (51) RNA alignment methods align sequences using predicted structure as auxiliary information, but these methods also have significant differences with TurboFold II.

For MAFFT, the X-INS-i option provides the capability for incorporating structural information in a multiple sequence alignment (MSA); hence forth, MAFFT refers to the program used with this option. To obtain a multiple sequence alignment, MAFFT first calculates pairwise structural alignments using either the SCARNA (65) or LaRA (66) methods. Using a guide tree and consistency score, an initial MSA is computed progressively from the pairwise structural alignments. This MSA is then iteratively refined to incorporate structural information represented as base pairing probabilities for each sequence computed using the McCaskill algorithm (39). The iterative refinement optimizes an alignment score that combines a weighted sum of pairs term (67) that assesses sequence conservation, a consistency term (68) that assesses consistency of the MSA with the pairwise alignments, and a ‘four-way consistency’ term that encourages alignment of nucleotides in the two sequences whose paired nucleotides are aligned. The ‘four-way’ consistency incorporates the structural information in the alignment.

While both MAFFT and TurboFold II iteratively incorporate structural information in computing an MSA, the approaches differ fundamentally. The TurboFold II iterations alternate between structural predictions (updating base pairing probabilities) and alignment predictions (updating alignment probabilities). Both the structural and alignment prediction steps utilize probabilistic models and exchange information as prior probabilities. TurboFold II also refines the pairwise sequence alignments using structural information, in contrast to MAFFT using structural information at the MSA refinement.

R-Coffee is a variant of T-Coffee (36). It starts by generating pairwise sequence alignments, called a library, and then estimates a MSA from the pairwise alignment library using the individually aligned nucleotide positions from the library as ‘weighted constraints’. RNA secondary structure information is also included in the refinement in the form of local base paring probabilities, which are calculated by RNAplfold (69,70).

In R-Coffee, the MSA is assembled from library of nucleotide alignments in a way that favors a 4-way-consistency, i.e. nucleotides are more likely to align if they align to common nucleotide in a third sequence and if they have high probability of base pairing with nucleotides that are also aligned in the library. Sequences are aligned pairwise (71) with a score that favors 4-way consistency, a tree is built (72), and the multiple alignment assembled (49).

A major difference between TurboFold II and both MAFFT and R-Coffee is that the match score in TurboFold II reflects the general similarity of base pairing conditions (being paired upstream, paired downstream, or unpaired) rather than restraints as being paired with particular nucleotides. The advantage of the match score is not to limit the potential alignment partners in too narrow a range. By combining with sequence identity in the HMM calculation, it can be useful to improve the overall alignments based on imperfect structure prediction, particularly at the beginning of the iterations.

### Scoring of prediction accuracy

For both predicted alignments and structures, sensitivity and positive predictive value (PPV) were calculated. For the alignment benchmark, sensitivity is the fraction of aligned nucleotide pairs in the database that are correctly predicted by the methods. PPV is the fraction of predicted aligned nucleotide pairs that also occur in the accepted alignment (53,73,74). For the secondary structure benchmark, sensitivity is the fraction of base pairs annotated in the database that are correctly predicted. PPV is the fraction of the predicted base pairs that also occur in the accepted structures in the database. Predicted base pairs are considered correct if a nucleotide either on 5′ or 3′ end of the correct base is off by one position (75). For instance, a predicted base pair ($i$, $j$) is correct if base pair ($i$, $j$), ($i$±1, $j$) or ($i$, $j$±1) exists in database. This is important because of uncertainty in the determination of secondary structure by comparative analysis (76) and also because of thermodynamic fluctuations of local structures (77–79). The scorer program of RNAstructure was used.

### Significance testing

To assess the statistical significance of the differences in sensitivity and PPV, paired t-tests were performed using R 3.0.2 (URL: http://www.R-project.org/) (80) between TurboFold II and each of the other methods (81). Alpha, the type I error rate, was set to 0.05. The figures summarizing the benchmarking results are annotated to indicate the results of the significance tests.

## RESULTS

### Algorithm overview

Fundamentally, TurboFold II is an extension of TurboFold (38), which takes multiple homologous RNA sequences as input and outputs estimated base pair probabilities, where the estimates for each sequence are informed by the other sequences. The main enhancement from TurboFold to TurboFold II is that, in the iterations, the pairwise posterior co-incidence probabilities for alignments are also updated, guided by estimated base pairing probabilities, and, upon completion of iterations, a multiple sequence alignment is obtained via the probabilistic consistency-based progressive alignment method of ProbCons (37). Just like TurboFold, TurboFold II does not enforce predictions into a single common structure, therefore, it is able to predict diverged structures for homologous sequences.

### Comparison to other programs

#### Alignment Prediction

**Figure 2.**
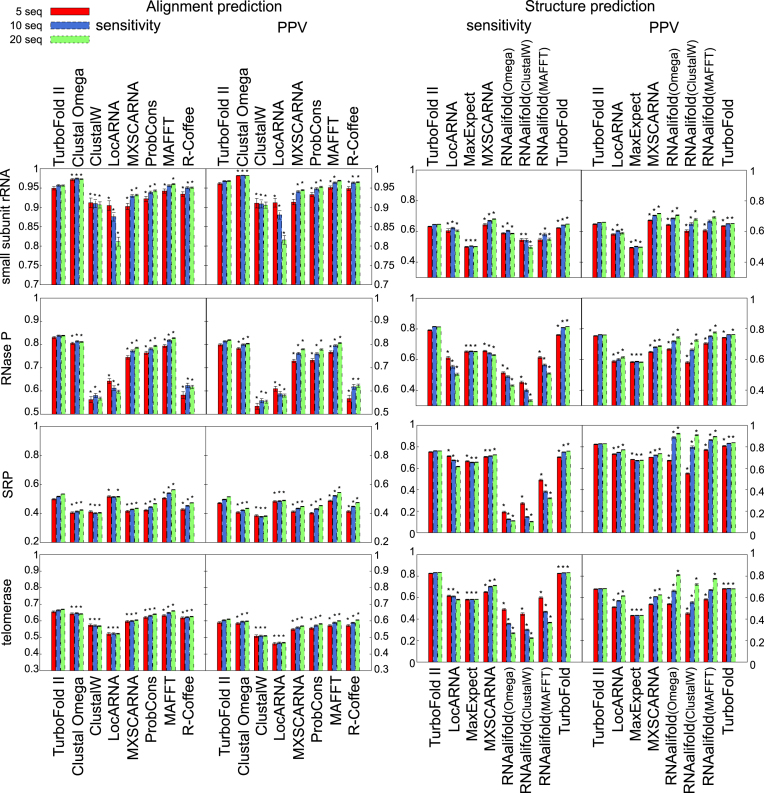
Sensitivity and PPV of alignment (left) and structure (right) predictions. Sensitivity and PPV of alignment predictions obtained by running the methods with 5, 10 or 20 input sequences on the small subunit rRNA, RNase P RNA, SRP RNA and telomerase RNA test datasets. The star (*) above the bar for a method indicates that the difference in sensitivity (or PPV) between the method and TurboFold II is statistically significant, as determined by a paired t-test. Numerical sensitivity and PPV values corresponding to the plots in the figures are provided in the Supplementary Materials in Tables S1 and S2 for alignment and structure prediction, respectively.

The accuracy of TurboFold II was compared to those of seven leading multiple alignment methods: Clustal Omega (1.2.1) (48), a method that uses HMM alignment that is based on the HHalign package (82) and guide tree computation that utilizes an enhanced version of mBed (83) and can cluster large numbers of sequences rapidly; ClustalW (2.1) (49), a method that is based on pairwise dynamic programing alignments (84) and a neighbor joining clustering algorithm (72); LocARNA (1.8.7) (22), a Sankoff-style structure-based alignment method that implements the algorithm of comparison of estimated base pairing probabilities that was proposed in PMcomp (35); MXSCARNA (2.1) (23), a structural-alignment method that progressively aligns potential stem candidates after removing the inconsistent stem components that are overlapping with others; ProbCons (1.12) (37), a method based on HMM-derived posterior probability and three-way probabilistic consistency; MAFFT (X-INS-i option) (50), a method that utilizes pairwise structural alignments calculated by SCARNA (65) and progressively combines them to create a multiple sequence alignment; and R-Coffee (51), an approach that extends T-Coffee's algorithm by refining the score of the pairwise nucleotide alignments by considering the predicted base pairing of nucleotides. Calculations were performed on 200 sets of 5, 10 and 20 homologous sequences of small subunit rRNA (58), RNase P RNA (60), SRP RNA (59) and telomerase RNA (61). All methods were run with default parameters. The results are shown in Figure 2.

With the exception of the small subunit rRNA family, TurboFold II had the highest sensitivity and PPV among the programs benchmarked. The pairwise sequence identities for the families used in the benchmarking are tabulated in the Supplementary Material, where the pairwise sequence identity is defined as the fraction of nucleotide positions for which the nucleotides are aligned and identical. The small subunit rRNA family sequences have the highest average pairwise sequence identity among all the families (Supplementary Figure S1), therefore, the sequence-based alignment methods tend to be more successful for those sequences. Sequence-identity-based methods, however, tend to perform poorly on families with low pairwise sequence similarity, including SRP and RNase P. Additional benchmarks of multiple sequence alignment by TurboFold II on the BRAliBase 2.0 dataset demonstrated that TurboFold II performed well, especially in the low sequence identity region (Supplementary Figure S3).

#### Structure Prediction

The secondary structure prediction results from TurboFold II over the test datasets were compared against leading secondary structure prediction methods: LocARNA (1.8.7) (22); RNAalifold (2.1.9) (62), a method that reads aligned RNA sequences and computes minimum free energy conserved structures as allowed by the input alignment; MXSCARNA (2.1) (23), which predicts a consensus structure by Rfold and input from ClustalW (2.1) (49); and TurboFold (38). MaxExpect (45,63), a single sequence structure prediction method, is used as a control calculation because it also predicts structure with the maximum expected accuracy algorithm, which is same as the mode chosen in TurboFold II and TurboFold. The required alignment input for RNAalifold was calculated by ClustalW, Clustal Omega (1.2.1) (48), or MAFFT (X-INS-i). The results are shown in Figure 2.

For each family, TurboFold II had a sensitivity and PPV comparable to TurboFold and performed well in comparison with other methods (Figure 2). Except for the small subunit rRNA family, TurboFold II and TurboFold are the top two methods when considering the average of sensitivity and PPV. Among the methods compared, MXSCARNA has the highest accuracy for the small subunit rRNA. The accuracy of RNAalifold depended on the alignment quality. For sequences of small subunit rRNA, RNase P RNA, and telomerase RNA, RNAalifold performs better structure predictions with input alignments from Clustal Omega and MAFFT than from ClustalW, which corresponded with the relative alignment accuracy of the methods (Figure 2).

## DISCUSSION

TurboFold offered a breakthrough by predicting conserved RNA secondary structures using probabilistic alignment information rather than fixed input alignments. It lacked, however, a mechanism for estimating the alignments using structural information. TurboFold II fills this lacuna by incorporating iterative refinement of the alignment probabilities in addition to that of the base pairing probabilities. This additional functionality is introduced in TurboFold II by using a match score function that represents the secondary structural similarity between two nucleotides (in two sequences) based on estimated base pairing probabilities. Thus, the computation of extrinsic information for structures also uses updated posterior co-incidence probabilities to re-estimate base pairing probabilities for each sequence. The final predicted alignment additionally benefits from the consistency transformation introduced by ProbCons (37). The pairwise comparison scores are used in progressive alignment to output a final multiple sequence alignment.

Structural alignment methods, like TurboFold II, take advantage of predicted structural information to inform sequence alignments. In contrast, sequence alignment methods rely solely on nucleotide identity, which is problematic because of the relatively poor sequence conservation compared to structure conservation in RNA.

As with other structural alignment tools, a limitation of TurboFold II is that its alignment accuracy heavily relies on the accuracy of secondary structure prediction. When a sequence has variable structure elements that are absent in the other input sequences, the extrinsic information computed from other sequences for the corresponding regions is not as useful as when there are similar structural elements in at least one other input sequence. These structural inserts are common in several RNA families, such as RNase P RNA and SRP RNA (77). A detailed example of such a case in RNase P is shown in Supplementary Figure S4, with the known secondary structures for five RNase P sequences, *Nocardioides albus, Propioniferax innocua, Salt Marsh A26, Mycobacterium tuberculosis* and *Lake Griffy A #8* in Supplementary Figure S4(a–e). The known structure for *Nocardioides albus* in Supplementary Figure S4(a) was different from other two structures *Propioniferax innocua* in Supplementary Figure S4(b) and *Salt Marsh A26* in Supplementary Figure S4(c), with a three-arm multibranch loop (helixes are marked by colors). On the other hand, structures for *Propioniferax innocua* and *Salt Marsh A26* contain a bulge loop in the corresponding position. Therefore, an inserted hairpin structure in *Nocardioides albus* makes the secondary structure different from those for *Propioniferax innocua* and *Salt Marsh A26*.

**Figure 3.**
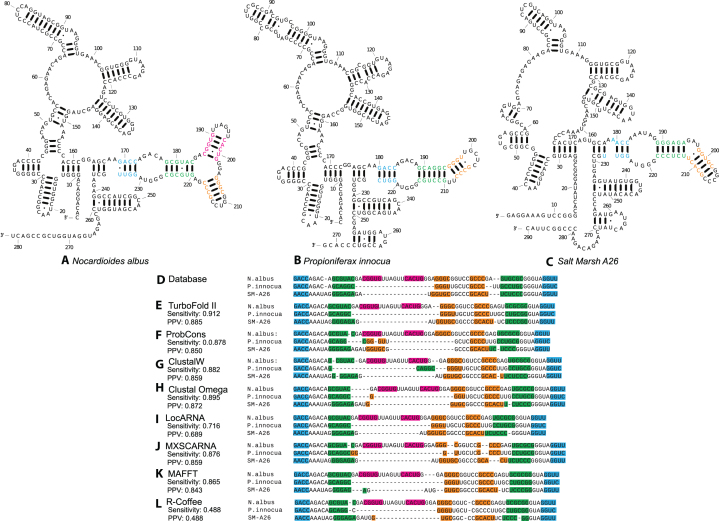
Predicted structures and alignments for *Nocardioides albus, Propioniferax innocua* and *Salt Marsh A26*. Structures for *Nocardioides albus* (**A**), *Propioniferax innocua* (**B**) and *Salt Marsh A26* (**C**) as predicted by TurboFold II. (**D**) Database alignments for *Nocardioides albus, Propioniferax innocua* and *Salt Marsh A26*. Alignments as predicted by TurboFold II (**E**), ProbCons (**F**), ClustalW (**G**), Clustal Omega (**H**), LocARNA (**I**), MXSCARNA (**J**), MAFFT (**K**) and R-Coffee (**L**). The alignment accuracy is indicated as sensitivity and PPV for each method. The colored nucleotides correspond to helices in database structures.

TurboFold II inherits the beneficial capability of TurboFold that allows variable structural elements within individual structures. For these RNase P sequences, the flexibility of the model of structural conservation is clear. Figure 3 (panels a–c) shows the structures for *Nocardioides albus, Propioniferax innocua* and *Salt Marsh A26* as predicted by TurboFold II. The multibranch loop and bulge loops are correctly predicted. Figure 3d shows the known alignment of nucleotides of the variable structure elements for *Nocardioides albus, Propioniferax innocua* and *Salt Marsh A26*. The nucleotides of the aligned helices alignments are colored according to their secondary structures. Figure 3 (panes e–l) shows the predicted sequence alignments and prediction accuracies for TurboFold II, ProbCons, ClustalW, Clustal Omega, LocARNA, MXSCARNA, MAFFT, and R-Coffee. The multiple sequence alignments output by TurboFold II achieved the highest prediction accuracy (both sensitivity and PPV) among all methods. The helix of the inserted structural domain (indicated by magenta coloring in Figure 3, panels a–c) in *Nocardioides albus* is correctly predicted as an insertion by TurboFold II, by two other structural alignment methods, LocARNA and MXSCARNA, and by the purely sequence-based method, Clustal Omega. Without the benefit of structural information, this helical region is aligned incorrectly with nucleotides in 5′-end of another helix in the ProbCons prediction and with nucleotides in 3′-end of another helix in the ClustalW prediction. Supplementary Figures S6–S13 in the Supplementary Materials show the complete predicted sequence alignments from TurboFold II, Clustal Omega, ClustalW, LocARNA and MXSCARNA, ProbCons, MAFFT and R-Coffee, respectively. Supplementary Figures S14–S20 show the predicted structures by TurboFold II, LocARNA, MaxExpect, MXSCARNA, RNAalifold (using Clustal Omega alignment), RNAalifold (using ClustalW alignment) and TurboFold, respectively.

**Figure 4.**
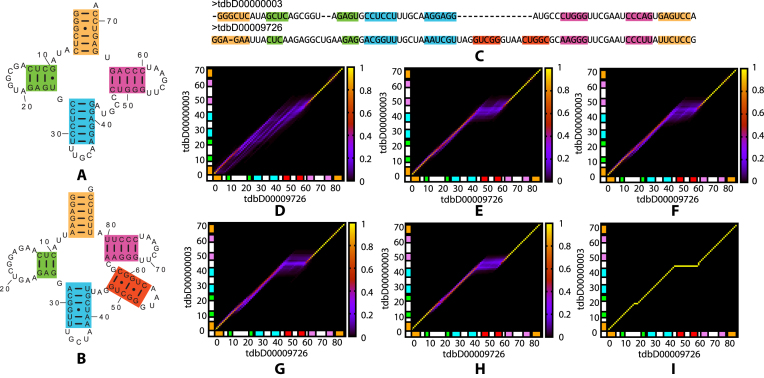
An example from the alignment of tRNA sequence homologs that illustrates how the update of posterior coincidence probabilities introduced in TurboFold II can improve alignments by incorporating structural information. tRNA structures of (**A**) *Halorubrum lacusprofundi* (tdbD00000003) and (**B**) *Streptococcus pneumoniae TIGR4* (tdbD00009726) (57,85) by TurboFold II with three other tRNAs. (**C**) Predicted alignment of the two sequences. The nucleotides in predicted helices are indicated by corresponding colors in both the alignment and the structures. (**D**) The posterior co-incidence probabilities calculated by pairwise HMM alignment. The co-incidence probabilities are color coded as shown by the adjacent key. (**E**) The posterior co-incidence probabilities of pairwise HMM alignment incorporating the match score. (**F**–**H**) Posterior co-incidence probabilities by incorporating match score after first (F), second (G) and third (H) iterations, respectively. (*I*) The alignment from the Sprinzl database (48,68). The colored blocks along the axes in the alignment probability plots (D–I) identify the nucleotides for helices shown in (A), (B) and (C).

TurboFold II uses a relatively simple match score scheme to incorporate structural information into HMM alignments so that the computational demands remain comparable to TurboFold. Although the match score does not distinguish between nucleotides in same structure components (5′ stem, 3′ stem or unpaired), by combining with pairwise HMM alignments and probabilistic constraints, the nucleotides with relatively high posterior co-incidence probabilities are aligned and incorrect alignments at the border of stem and loop regions are excluded. An example of such a case in tRNA is shown in Figure 4. Figure 4A and D depicts the predicted structures of two homologous tRNA sequences *Halorubrum lacusprofundi* (database ID: tdbD00000003, anticodon: UGC, amino acid: Ala) and *Streptococcus pneumoniae* TIGR4 (database ID: tdbD00009726, anticodon: GCU, amino acid: Ser), respectively. Figure 4C is the predicted alignment. Compared with the relatively diffuse posterior co-incidence probabilities for the variable hairpin loop structure from the initial pairwise HMM alignment (Figure 4D), the posterior co-incidence probabilities obtained with TurboFold II (Figure 4H) are sharper for the second hairpin loop structure and the variable region is more distinguishable as an insertion in the second sequence. The gradually change in the posterior co-incidence probabilities during the iterations (Figure 4E–H) shows that distribution of the probability mass becomes more consistent with the database alignment (Figure 4I) as the iterations proceed.

TurboFold II now iteratively refines multiple sequence alignments and estimated secondary structures, estimating both nucleotide alignment probabilities for sequence pairs and base pairing probabilities for base pairs. Dynamic programing algorithms accomplish both steps, but the simultaneous folding and alignment problem is avoided, and thus TurboFold II accomplishes sequence alignment and structure prediction with much better overall scaling, *O*(*H^2^N^2^ + HN^3^*) for *H* sequences of average length *N*. The time performance on select sequence families is provided in the Supplementary Materials in Table S3.

## DATA AVAILABILITY

TurboFold II is a component of the RNAstructure software package and is available for download from http://rna.urmc.rochester.edu. Source code and binaries are available. Additionally, a C++ class is available for incorporating TurboFold II into other software packages.

## Supplementary Material

Supplementary Data

## References

[1] Stark B.C., Kole R., Bowman E.J., Altman S. Ribonuclease P: an enzyme with an essential RNA component. Proc. Natl. Acad. Sci. U.S.A. 1978; 75:3717–3721. 358197 10.1073/pnas.75.8.3717 PMC392857

[2] Cech T.R., Zaug A.J., Grabowski P.J. In vitro splicing of the ribosomal RNA precursor of Tetrahymena: involvement of a guanosine nucleotide in the excision of the intervening sequence. Cell. 1981; 27:487–496. 6101203 10.1016/0092-8674(81)90390-1

[3] Doudna J.A., Cech T.R. The chemical repertoire of natural ribozymes. Nature. 2002; 418:222–228. 12110898 10.1038/418222a

[4] Griffiths-Jones S. Annotating noncoding RNA genes. Annu. Rev. Genomics Hum. Genet. 2007; 8:279–298. 17506659 10.1146/annurev.genom.8.080706.092419

[5] Eddy S.R. Non-coding RNA genes and the modern RNA world. Nat. Rev. Genet. 2001; 2:919–929. 11733745 10.1038/35103511

[6] Mathews D.H., Turner D.H. Prediction of RNA secondary structure by free energy minimization. Curr. Opin. Struct. Biol. 2006; 16:270–278. 16713706 10.1016/j.sbi.2006.05.010

[7] Seetin M.G., Mathews D.H. RNA structure prediction: an overview of methods. Methods. Mol. Biol. 2012; 905:99–122. 22736001 10.1007/978-1-61779-949-5_8

[8] Hofacker I.L. Energy-directed RNA structure prediction. Methods. Mol. Biol. 2014; 1097:71–84. 24639155 10.1007/978-1-62703-709-9_4

[9] Havgaard J.H., Gorodkin J. RNA structural alignments, part I: Sankoff-based approaches for structural alignments. Methods. Mol. Biol. 2014; 1097:275–290. 24639164 10.1007/978-1-62703-709-9_13

[10] Asai K., Hamada M. RNA structural alignments, part II: non-Sankoff approaches for structural alignments. Methods. Mol. Biol. 2014; 1097:291–301. 24639165 10.1007/978-1-62703-709-9_14

[11] Hua L., Song Y., Kim N., Laing C., Wang J.T., Schlick T. CHSalign: a web server that builds upon junction-explorer and RNAJAG for pairwise alignment of RNA secondary structures with coaxial helical stacking. PLoS One. 2016; 11:e0147097. 26789998 10.1371/journal.pone.0147097 PMC4720362

[12] Dror O., Nussinov R., Wolfson H. ARTS: alignment of RNA tertiary structures. Bioinformatics. 2005; 21(Suppl. 2):ii47–ii53. 16204124 10.1093/bioinformatics/bti1108

[13] Poch O., Sauvaget I., Delarue M., Tordo N. Identification of four conserved motifs among the RNA-dependent polymerase encoding elements. EMBO J. 1989; 8:3867–3874. 2555175 10.1002/j.1460-2075.1989.tb08565.x PMC402075

[14] Brown E.A., Zhang H., Ping L.H., Lemon S.M. Secondary structure of the 5′ nontranslated regions of hepatitis C virus and pestivirus genomic RNAs. Nucleic Acids Res. 1992; 20:5041–5045. 1329037 10.1093/nar/20.19.5041 PMC334281

[15] Ritz J., Martin J.S., Laederach A. Evolutionary evidence for alternative structure in RNA sequence co-variation. PLoS Comput. Biol. 2013; 9:e1003152. 23935473 10.1371/journal.pcbi.1003152 PMC3723493

[16] Hwang J.S., Lee J.S., Goo T.W., Yun E.Y., Sohn H.R., Kim H.R., Kwon O.Y. Molecular genetic relationships between Bombycidae and Saturniidae based on the mitochondria DNA encoding of large and small rRNA. Genet. Anal. 1999; 15:223–228. 10609758 10.1016/s1050-3862(99)00008-x

[17] Gruber A.R., Findeiss S., Washietl S., Hofacker I.L., Stadler P.F. RNAz 2.0: improved noncoding RNA detection. Pac. Symp. Biocomput. 2010; 69–79. 19908359

[18] Fu Y., Xu Z.Z., Lu Z.J., Zhao S., Mathews D.H. Discovery of novel ncRNA sequences in multiple genome alignments on the basis of conserved and stable secondary structures. PLoS One. 2015; 10:e0130200. 26075601 10.1371/journal.pone.0130200 PMC4468099

[19] Henikoff S., Henikoff J.G. Amino acid substitution matrices from protein blocks. Proc. Natl. Acad. Sci. U.S.A. 1992; 89:10915–10919. 1438297 10.1073/pnas.89.22.10915 PMC50453

[20] Pace N.R., Thomas B.C., Woese C.R. Gesteland RF, Cech TR, Atkins JF The RNA World. 1999; 2nd edn, NY: Cold Spring Harbor Laboratory Press 113–141.

[21] van Nimwegen E., Crutchfield J.P., Huynen M. Neutral evolution of mutational robustness. Proc. Natl. Acad. Sci. U.S.A. 1999; 96:9716–9720. 10449760 10.1073/pnas.96.17.9716 PMC22276

[22] Will S., Reiche K., Hofacker I.L., Stadler P.F., Backofen R. Inferring noncoding RNA families and classes by means of genome-scale structure-based clustering. PLoS Comput. Biol. 2007; 3:e65. 17432929 10.1371/journal.pcbi.0030065 PMC1851984

[23] Tabei Y., Kiryu H., Kin T., Asai K. A fast structural multiple alignment method for long RNA sequences. BMC Bioinformatics. 2008; 9:33. 18215258 10.1186/1471-2105-9-33 PMC2375124

[24] Xu Z., Mathews D.H. Multilign: an algorithm to predict secondary structures conserved in multiple RNA sequences. Bioinformatics. 2011; 27:626–632. 21193521 10.1093/bioinformatics/btq726 PMC3042186

[25] Havgaard J.H., Torarinsson E., Gorodkin J. Fast pairwise structural RNA alignments by pruning of the dynamical programming matrix. PLoS Comput. Biol. 2007; 3:1896–1908. 17937495 10.1371/journal.pcbi.0030193 PMC2014794

[26] Sankoff D. Simultaneous solution of the RNA folding, alignment and protosequence problems. SIAM J. Appl. Math. 1985; 45:810–825.

[27] Mathews D.H., Turner D.H. Dynalign: an algorithm for finding the secondary structure common to two RNA sequences. J. Mol. Biol. 2002; 317:191–203. 11902836 10.1006/jmbi.2001.5351

[28] Harmanci A.O., Sharma G., Mathews D.H. Efficient pairwise RNA structure prediction using probabilistic alignment constraints in Dynalign. BMC Bioinformatics. 2007; 8:130. 17445273 10.1186/1471-2105-8-130 PMC1868766

[29] Will S., Otto C., Miladi M., Mohl M., Backofen R. SPARSE: quadratic time simultaneous alignment and folding of RNAs without sequence-based heuristics. Bioinformatics. 2015; 31:2489–2496. 25838465 10.1093/bioinformatics/btv185 PMC4514930

[30] Uzilov A.V., Keegan J.M., Mathews D.H. Detection of non-coding RNAs on the basis of predicted secondary structure formation free energy change. BMC Bioinformatics. 2006; 7:173. 16566836 10.1186/1471-2105-7-173 PMC1570369

[31] Harmanci A.O., Sharma G., Mathews D.H. PARTS: probabilistic alignment for RNA joinT secondary structure prediction. Nucleic Acids Res. 2008; 36:2406–2417. 18304945 10.1093/nar/gkn043 PMC2367733

[32] Hofacker I.L., Stadler P.F. Bubak M, vanAlbada GD, Sloot PMA, Dongarra JJ Computational Science - ICCS 2004, volume 3039 of Lecture Notes in Computer Science. 2004; 6–9:Kraków 728–735.

[33] Dalli D., Wilm A., Mainz I., Steger G. STRAL: progressive alignment of non-coding RNA using base pairing probability vectors in quadratic time. Bioinformatics. 2006; 22:1593–1599. 16613908 10.1093/bioinformatics/btl142

[34] Torarinsson E., Havgaard J.H., Gorodkin J. Multiple structural alignment and clustering of RNA sequences. Bioinformatics. 2007; 23:926–932. 17324941 10.1093/bioinformatics/btm049

[35] Hofacker I.L., Bernhart S.H., Stadler P.F. Alignment of RNA base pairing probability matrices. Bioinformatics. 2004; 20:2222–2227. 15073017 10.1093/bioinformatics/bth229

[36] Notredame C., Higgins D.G., Heringa J. T-Coffee: A novel method for fast and accurate multiple sequence alignment. J. Mol. Biol. 2000; 302:205–217. 10964570 10.1006/jmbi.2000.4042

[37] Do C.B., Mahabhashyam M.S., Brudno M., Batzoglou S. ProbCons: probabilistic consistency-based multiple sequence alignment. Genome Res. 2005; 15:330–340. 15687296 10.1101/gr.2821705 PMC546535

[38] Harmanci A.O., Sharma G., Mathews D.H. TurboFold: iterative probabilistic estimation of secondary structures for multiple RNA sequences. BMC Bioinformatics. 2011; 12:108. 21507242 10.1186/1471-2105-12-108 PMC3120699

[39] McCaskill J.S. The equilibrium partition function and base pair binding probabilities for RNA secondary structure. Biopolymers. 1990; 29:1105–1119. 1695107 10.1002/bip.360290621

[40] Mathews D.H., Disney M.D., Childs J.L., Schroeder S.J., Zuker M., Turner D.H. Incorporating chemical modification constraints into a dynamic programming algorithm for prediction of RNA secondary structure. Proc. Natl. Acad. Sci. U.S.A. 2004; 101:7287–7292. 15123812 10.1073/pnas.0401799101 PMC409911

[41] Turner D.H., Mathews D.H. NNDB: the nearest neighbor parameter database for predicting stability of nucleic acid secondary structure. Nucleic Acids Res. 2010; 38:D280–282. 19880381 10.1093/nar/gkp892 PMC2808915

[42] Durbin R., Eddy S.R., Krogh A., Mitchison G. Biological Sequence Analysis: Probabilistic Models of Proteins and Nucleic Acids. 1998; Cambridge: Cambridge University Press.

[43] Knudsen B., Hein J. Pfold: RNA secondary structure prediction using stochastic context-free grammars. Nucleic Acids Res. 2003; 31:3423–3428. 12824339 10.1093/nar/gkg614 PMC169020

[44] Do C.B., Woods D.A., Batzoglou S. CONTRAfold: RNA secondary structure prediction without physics-based models. Bioinformatics. 2006; 22:e90–e98. 16873527 10.1093/bioinformatics/btl246

[45] Lu Z.J., Gloor J.W., Mathews D.H. Improved RNA secondary structure prediction by maximizing expected pair accuracy. RNA. 2009; 15:1805–1813. 19703939 10.1261/rna.1643609 PMC2743040

[46] Bellaousov S., Mathews D.H. ProbKnot: fast prediction of RNA secondary structure including pseudoknots. RNA. 2010; 16:1870–1880. 20699301 10.1261/rna.2125310 PMC2941096

[47] Seetin M.G., Mathews D.H. TurboKnot: rapid prediction of conserved RNA secondary structures including pseudoknots. Bioinformatics. 2012; 28:792–798. 22285566 10.1093/bioinformatics/bts044 PMC3307117

[48] Sievers F., Wilm A., Dineen D., Gibson T.J., Karplus K., Li W., Lopez R., McWilliam H., Remmert M., Soding J. Fast, scalable generation of high-quality protein multiple sequence alignments using Clustal Omega. Mol. Syst. Biol. 2011; 7:539. 21988835 10.1038/msb.2011.75 PMC3261699

[49] Larkin M.A., Blackshields G., Brown N.P., Chenna R., McGettigan P.A., McWilliam H., Valentin F., Wallace I.M., Wilm A., Lopez R. Clustal W and Clustal X version 2.0. Bioinformatics. 2007; 23:2947–2948. 17846036 10.1093/bioinformatics/btm404

[50] Katoh K., Toh H. Improved accuracy of multiple ncRNA alignment by incorporating structural information into a MAFFT-based framework. BMC Bioinformatics. 2008; 9:212. 18439255 10.1186/1471-2105-9-212 PMC2387179

[51] Wilm A., Higgins D.G., Notredame C. R-Coffee: a method for multiple alignment of non-coding RNA. Nucleic Acids Res. 2008; 36:e52. 18420654 10.1093/nar/gkn174 PMC2396437

[52] Mathews D.H. Using an RNA secondary structure partition function to determine confidence in base pairs predicted by free energy minimization. RNA. 2004; 10:1178–1190. 15272118 10.1261/rna.7650904 PMC1370608

[53] Gardner P.P., Wilm A., Washietl S. A benchmark of multiple sequence alignment programs upon structural RNAs. Nucleic Acids Res. 2005; 33:2433–2439. 15860779 10.1093/nar/gki541 PMC1087786

[54] Szymanski M., Barciszewska M.Z., Erdmann V.A., Barciszewski J. 5S ribosomal RNA database. Nucleic Acids Res. 2002; 30:176–178. 11752286 10.1093/nar/30.1.176 PMC99124

[55] Zhou Y., Lu C., Wu Q.J., Wang Y., Sun Z.T., Deng J.C., Zhang Y. GISSD: group I intron sequence and structure database. Nucleic Acids Res. 2008; 36:D31–D37. 17942415 10.1093/nar/gkm766 PMC2238919

[56] Zwieb C., Gorodkin J., Knudsen B., Burks J., Wower J. tmRDB (tmRNA database). Nucleic Acids Res. 2003; 31:446–447. 12520048 10.1093/nar/gkg019 PMC165466

[57] Juhling F., Morl M., Hartmann R.K., Sprinzl M., Stadler P.F., Putz J. tRNAdb 2009: compilation of tRNA sequences and tRNA genes. Nucleic Acids Res. 2009; 37:D159–162. 18957446 10.1093/nar/gkn772 PMC2686557

[58] Cannone J.J., Subramanian S., Schnare M.N., Collett J.R., D'Souza L.M., Du Y., Feng B., Lin N., Madabusi L.V., Muller K.M. The comparative RNA web (CRW) site: an online database of comparative sequence and structure information for ribosomal, intron, and other RNAs. BMC Bioinformatics. 2002; 3:2. 11869452 10.1186/1471-2105-3-2 PMC65690

[59] Rosenblad M.A., Gorodkin J., Knudsen B., Zwieb C., Samuelsson T. SRPDB: signal recognition particle database. Nucleic Acids Res. 2003; 31:363–364. 12520023 10.1093/nar/gkg107 PMC165554

[60] Brown J.W. The ribonuclease P database. Nucleic Acids Res. 1999; 27:314. 9847214 10.1093/nar/27.1.314 PMC148169

[61] Nawrocki E.P., Burge S.W., Bateman A., Daub J., Eberhardt R.Y., Eddy S.R., Floden E.W., Gardner P.P., Jones T.A., Tate J. Rfam 12.0: updates to the RNA families database. Nucleic Acids Res. 2015; 43:D130–D137. 25392425 10.1093/nar/gku1063 PMC4383904

[62] Bernhart S.H., Hofacker I.L., Will S., Gruber A.R., Stadler P.F. RNAalifold: improved consensus structure prediction for RNA alignments. BMC Bioinformatics. 2008; 9:474. 19014431 10.1186/1471-2105-9-474 PMC2621365

[63] Reuter J.S., Mathews D.H. RNAstructure: software for RNA secondary structure prediction and analysis. BMC Bioinformatics. 2010; 11:129. 20230624 10.1186/1471-2105-11-129 PMC2984261

[64] Lowes B., Chauve C., Ponty Y., Giegerich R. The BRaliBase dent-a tale of benchmark design and interpretation. Brief Bioinform. 2017; 18:306–311. 26984616 10.1093/bib/bbw022 PMC5444242

[65] Tabei Y., Tsuda K., Kin T., Asai K. SCARNA: fast and accurate structural alignment of RNA sequences by matching fixed-length stem fragments. Bioinformatics. 2006; 22:1723–1729. 16690634 10.1093/bioinformatics/btl177

[66] Bauer M., Klau G.W., Reinert K. Accurate multiple sequence-structure alignment of RNA sequences using combinatorial optimization. BMC Bioinformatics. 2007; 8:271. 17662141 10.1186/1471-2105-8-271 PMC1955456

[67] Gotoh O. A weighting system and algorithm for aligning many phylogenetically related sequences. Comput. Appl. Biosci. 1995; 11:543–551. 8590178 10.1093/bioinformatics/11.5.543

[68] Notredame C., Holm L., Higgins D.G. COFFEE: an objective function for multiple sequence alignments. Bioinformatics. 1998; 14:407–422. 9682054 10.1093/bioinformatics/14.5.407

[69] Bernhart S.H., Hofacker I.L., Stadler P.F. Local RNA base pairing probabilities in large sequences. Bioinformatics. 2006; 22:614–615. 16368769 10.1093/bioinformatics/btk014

[70] Bompfunewerer A.F., Backofen R., Bernhart S.H., Hertel J., Hofacker I.L., Stadler P.F., Will S. Variations on RNA folding and alignment: lessons from Benasque. J, Math Biol. 2008; 56:129–144. 17611759 10.1007/s00285-007-0107-5

[71] Myers E.W., Miller W. Optimal alignments in linear space. Comput. Appl. Biosci. 1988; 4:11–17. 3382986 10.1093/bioinformatics/4.1.11

[72] Saitou N., Nei M. The neighbor-joining method: a new method for reconstructing phylogenetic trees. Mol. Biol. Evol. 1987; 4:406–425. 3447015 10.1093/oxfordjournals.molbev.a040454

[73] Eddy S.R. SQUID (computer software) http://www.squid-cache.org/.

[74] Wilm A., Mainz I., Steger G. An enhanced RNA alignment benchmark for sequence alignment programs. Algorithms Mol. Biol. 2006; 1:19. 17062125 10.1186/1748-7188-1-19 PMC1635699

[75] Mathews D.H., Sabina J., Zuker M., Turner D.H. Expanded sequence dependence of thermodynamic parameters improves prediction of RNA secondary structure. J. Mol. Biol. 1999; 288:911–940. 10329189 10.1006/jmbi.1999.2700

[76] Gutell R.R., Lee J.C., Cannone J.J. The accuracy of ribosomal RNA comparative structure models. Curr. Opin. Struct. Biol. 2002; 12:301–310. 12127448 10.1016/s0959-440x(02)00339-1

[77] Fu Y., Sharma G., Mathews D.H. Dynalign II: common secondary structure prediction for RNA homologs with domain insertions. Nucleic Acids Res. 2014; 42:13939–13948. 25416799 10.1093/nar/gku1172 PMC4267632

[78] Znosko B.M., Silvestri S.B., Volkman H., Boswell B., Serra M.J. Thermodynamic parameters for an expanded nearest-neighbor model for the formation of RNA duplexes with single nucleotide bulges. Biochemistry. 2002; 41:10406–10417. 12173927 10.1021/bi025781q

[79] Woodson S.A., Crothers D.M. Proton nuclear magnetic resonance studies on bulge-containing DNA oligonucleotides from a mutational hot-spot sequence. Biochemistry. 1987; 26:904–912. 3567151 10.1021/bi00377a035

[80] Development Core Team R R: A language and environment for statistical computing. R Foundation for Statistical Computing. 2013; Vienna.

[81] Xu Z., Almudevar A., Mathews D.H. Statistical evaluation of improvement in RNA secondary structure prediction. Nucleic Acids Res. 2012; 40:e26. 22139940 10.1093/nar/gkr1081 PMC3287165

[82] Soding J. Protein homology detection by HMM-HMM comparison. Bioinformatics. 2005; 21:951–960. 15531603 10.1093/bioinformatics/bti125

[83] Blackshields G., Sievers F., Shi W., Wilm A., Higgins D.G. Sequence embedding for fast construction of guide trees for multiple sequence alignment. Algorithms Mol. Biol. 2010; 5:21. 20470396 10.1186/1748-7188-5-21 PMC2893182

[84] Myers E.W., Miller W. Optimal alignments in linear space. Comput. Appl. Biosci. 1988; 4:11–17. 3382986 10.1093/bioinformatics/4.1.11

[85] Sprinzl M., Vassilenko K.S. Compilation of tRNA sequences and sequences of tRNA genes. Nucleic Acids Res. 2005; 33:D139–D140. 15608164 10.1093/nar/gki012 PMC539966

