# Supplementary material for: TurboFold II: RNA structural alignment and secondary structure prediction informed by multiple homologs
Source: Nucleic Acids Res. 2017 Sep 28;45(20):11570–81. doi: 10.1093/nar/gkx815 (PMC5714223; doi:10.1093/nar/gkx815)
Supplement: Supplementary Data [file gkx815_supp.pdf]

Supplementary information for

"TurboFold II: RNA Secondary Structure Prediction and Structural Alignment Informed by Multiple Homologs"

Zhen Tan, Yinghan Fu, Gaurav Sharma, and David H. Mathews

Details of pairwise sequence identity (Section 1), database used in parameter optimization and testing (Section 2), parameter optimization methods (Section 3), sequences being used in parameter optimization and benchmarking (Section 4), tables of values about method performance on alignment and structure predictions (Section 5), benchmark of TurboFold II on BRAliBase 2.0 (Section 6) and accepted database structures and alignments for the example in the Discussion (*Nocardioides albus*, *Propioniferax innocua*, and *Salt Marsh A26(12)*) (section 7) are provided.

Section 1. Pairwise sequence identity:

The pairwise sequence identity between two sequences is computed as the fraction of nucleotide positions for which the nucleotides are aligned and identical over the sum of aligned and inserted positions between the two sequences for the maximum likelihood alignment between the sequences. The sequence identity is computed with a pairwise alignment HMM (1,2). In Figure S1, histograms of pairwise sequence identity are provided for the datasets used in the benchmarks for each individual family: the 5S small subunit rRNA, group I intron RNA, tmRNA, tRNA, small subunit rRNA, RNase P RNA, SRP RNA, and telomerase RNA.

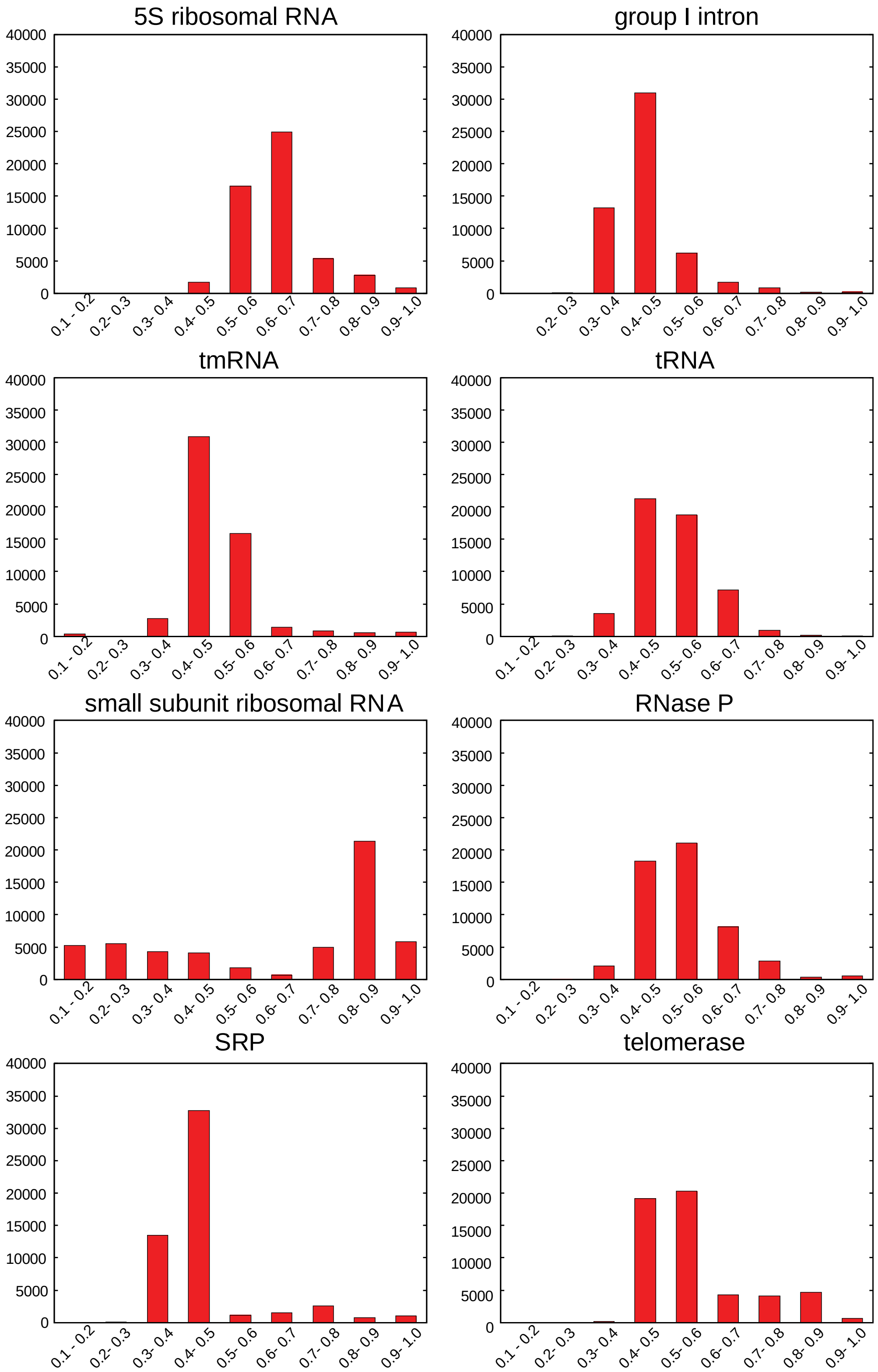

Figure S1. **Histograms for pairwise sequence identity for the datasets used in the benchmarks by each individual family: 5S rRNA, group I intron RNA, tmRNA, tRNA, small subunit rRNA, RNase P RNA, SRP RNA, and telomerase RNA.**

Section 2. Database: RNAStralign

For parameter optimization and benchmarking, an RNA alignment and structure database, named RNAStralign, was aggregated from available online databases of RNA structure and alignment. Structures for each family in RNAStralign are categorized into homologous families based on the classifications in the original databases. If available, further categorization into subfamilies was also included in RNAStralign. Only sequences with known alignments and secondary structures were included. The families included are:

1. Small subunit ribosomal RNA (16S rRNA) family

Small subunit rRNA structures and alignments were downloaded from the Comparative RNA Web Site (3).

2. 5S ribosomal RNA (5S rRNA) family

5S rRNA structures and alignments were downloaded from the 5S Ribosomal RNA Database (4).

3. Group I intron family

Group I intron structures and alignments were downloaded from the Group I Intron Sequence and Structure Database (GISSD) (5). Structures with P1 and P2 domain competition are represented by two structure files which either have P1 or P10 domain and with "_p1" or "_p10" added to the end of their file names.

4. RNase P family

RNase P structures and alignments were downloaded from the RNase P Database (6). Only 7 subfamilies with at least two full length structures (Bacterial Type A RNA, Bacterial Type B RNA, Archaeal RNA, Nuclear RNA, Plastid RNA, Vertibrate Nuclear RNA and Yeast Nuclear RNA) are kept in our database.

5. Signal recognition particle RNA (SRP RNA) family

SRP RNA structures and alignments were downloaded from the Signal Recognition Particle Database (SRPDB) (7).

6. Telomerase RNA family

Telomerase RNA structures and alignment were downloaded from the Rfam database (8) RF00024 family.

7. Transfer-messenger RNA family

Transfer-messenger RNA structures and alignment were downloaded from tmRNA Database (9).

8. Transfer RNA (tRNA) family

tRNA structures and alignments were downloaded from tRNAdb 2009 (10). Because the structures in this database do not include variable loops, covariance models of RNA secondary structure (COVE) (http://eddylab.org/software.html) were used to annotate the variable loop base pairs.

Section 3. Parameter optimization

The match scores for pairwise nucleotides *i* and *k* in two sequences are represented as

$$\rho(i,k) = \alpha_1\left(\sqrt{P_{<}^{m}(i)P_{<}^{n}(k)} + \sqrt{P_{>}^{m}(i)P_{>}^{n}(k)}\right) + \alpha_2\left(\sqrt{P_{\circ}^{m}(i)P_{\circ}^{n}(k)}\right) + \alpha_3$$

To train the three parameters in the match score scheme (α_1, α_2 and α_3), 40 groups of input sequences formed by 3, 5 and 7 homologous sequences, were randomly chosen from the 5S ribosome RNA (Eubacteria subfamily), group I intron (IC1 subfamily), tmRNA, and tRNA from the database RNAStralign.

A grid search was performed to find optimal parameter values. The search range for α_1 and α_2 were both by values of 0, 0.6, 0.8, 1.0, 2.0, 3.0, 4.0, and 5.0. α_3 was searched by values of 0, 0.3, 0.5, 0.7, and 1.0. Figure S2 shows the weighted accuracy of alignment and structure predictions by different parameters. The weighted accuracy is the product of sensitivity and positive predicted value (PPV) of averaged pairwise alignments predictions and averaged structure predictions by the number of input sequences. The sensitivity and PPV calculation are described in the Methods section of the paper. The highest accuracy is achieved by α_1 = 1.0, α_2 = 0.8 and α_3 = 0.5, which were used as the default for the TurboFold II.

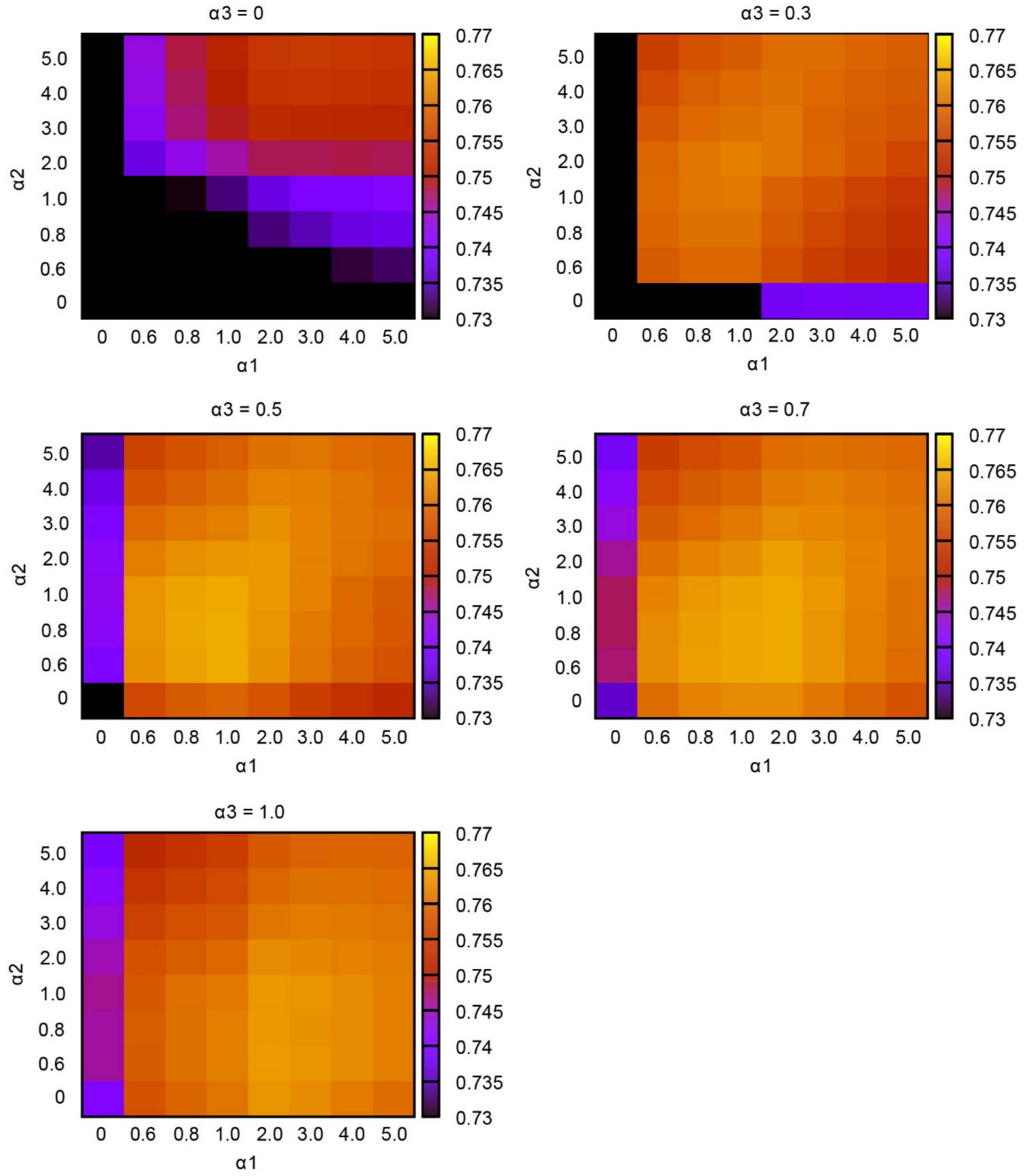

Figure S2. **Grid search plots for parameters α1, α2 and α3 in match score function.** The heat map keys on the right side show that the grid cells with higher values are represented in lighter color.

Section 4. Sequences used in parameters optimization and benchmarking

Sequences were separated in 3, 5, 7, 10, and 20 input sequences for each family (parameter optimization: 5S ribosome RNA (Eubacteria subfamily), group I intron (IC1 subfamily), tmRNA, and tRNA; benchmarking: small subunit ribosomal RNA (Alphaproteobacteria subfamily), SRP RNA (Protozoan subfamily), RNase P RNA (A bacteria subfamily) and telomerase RNA). Specific sequences lists are provided in “TurboFoldII_SelectedSequenceList.zip”.

Section 5. Performance of alignment methods on benchmarking dataset

Table S1. Alignment prediction sensitivity and PPV for each method on each dataset:

	**small subunit rRNA**					
	5 sequences		10 sequences		20 sequences	
	sensitivity	PPV	sensitivity	PPV	sensitivity	PPV
**TurboFold II**	0.949	0.961	0.957	0.968	0.956	0.968
**Clustal Omega**	**0.972**	**0.982**	**0.975**	**0.983**	**0.973**	**0.982**
**ClustalW**	0.912	0.911	0.910	0.909	0.906	0.906
**LocARNA**	0.905	0.912	0.876	0.880	0.811	0.816
**MXSCARNA**	0.902	0.914	0.929	0.941	0.932	0.945
**ProbCons**	0.922	0.933	0.939	0.948	0.943	0.953
**MAFFT**	0.942	0.951	0.956	0.964	0.960	0.969
**R-Coffee**	0.934	0.949	0.951	0.964	0.951	0.965

	**RNase P**					
	5 sequences		10 sequences		20 sequences	
	sensitivity	PPV	sensitivity	PPV	sensitivity	PPV
**TurboFold II**	**0.830**	**0.799**	**0.839**	**0.814**	**0.839**	**0.819**
**Clustal Omega**	0.805	0.783	0.814	0.800	0.811	0.804
**ClustalW**	0.563	0.534	0.581	0.558	0.567	0.554
**LocARNA**	0.643	0.610	0.612	0.588	0.597	0.580
**MXSCARNA**	0.745	0.729	0.772	0.762	0.786	0.780
**ProbCons**	0.764	0.732	0.782	0.760	0.794	0.778
**MAFFT**	0.794	0.767	0.818	0.796	0.828	0.806
**R-Coffee**	0.582	0.567	0.624	0.618	0.618	0.623

	**SRP RNA**					
	5 sequences		10 sequences		20 sequences	
	sensitivity	PPV	sensitivity	PPV	sensitivity	PPV
**TurboFold II**	0.498	0.471	0.517	0.497	0.533	0.514
**Clustal Omega**	0.404	0.407	0.413	0.422	0.423	0.434
**ClustalW**	0.411	0.384	0.401	0.377	0.404	0.381
**LocARNA**	**0.516**	0.483	0.513	0.485	0.515	0.489
**MXSCARNA**	0.415	0.411	0.427	0.434	0.435	0.448
**ProbCons**	0.422	0.402	0.444	0.431	0.467	0.453
**MAFFT**	0.505	**0.486**	**0.540**	**0.522**	**0.563**	**0.544**
**R-Coffee**	0.427	0.413	0.453	0.447	0.473	0.475

	telomerase					
	5 sequences		10 sequences		20 sequences	
	sensitivity	PPV	sensitivity	PPV	sensitivity	PPV
**TurboFold II**	**0.654**	**0.589**	**0.664**	**0.604**	**0.670**	**0.610**
**Clustal Omega**	0.642	0.585	0.647	0.596	0.642	0.598
**ClustalW**	0.574	0.508	0.571	0.509	0.568	0.510
**LocARNA**	0.522	0.462	0.524	0.467	0.523	0.468
**MXSCARNA**	0.596	0.549	0.600	0.560	0.605	0.569
**ProbCons**	0.621	0.555	0.631	0.572	0.640	0.582
**MAFFT**	0.632	0.571	0.649	0.589	0.660	0.600
**R-Coffee**	0.618	0.572	0.623	0.589	0.626	0.605

Table S2: Structure prediction sensitivity and PPV for each method on each dataset:

	small subunit rRNA					
	5 sequences		10 sequences		20 sequences	
	sensitivity	PPV	sensitivity	PPV	sensitivity	PPV
**TurboFold II**	0.632	0.648	0.645	0.658	0.649	0.657
**LocARNA**	0.605	0.582	0.622	0.605	0.604	0.589
**MaxExpect**	0.499	0.495	0.505	0.503	0.501	0.498
**MXSCARNA**	**0.644**	**0.673**	**0.670**	**0.705**	**0.680**	**0.718**
**RNAalifold (Clustal Omega)**	0.588	0.644	0.605	0.686	0.586	0.706
**RNAalifold (ClustalW)**	0.544	0.603	0.542	0.650	0.498	0.680
**RNAalifold (MAFFT)**	0.545	0.607	0.580	0.668	0.548	0.693
**TurboFold**	0.623	0.637	0.641	0.651	0.648	0.652

	RNase P					
	5 sequences		10 sequences		20 sequences	
	sensitivity	PPV	sensitivity	PPV	sensitivity	PPV
**TurboFold II**	**0.792**	**0.754**	**0.815**	0.761	**0.823**	0.763
**LocARNA**	0.612	0.587	0.554	0.600	0.505	0.614
**MaxExpect**	0.652	0.583	0.654	0.586	0.653	0.582
**MXSCARNA**	0.656	0.649	0.642	0.679	0.630	0.687
**RNAalifold (Clustal Omega)**	0.514	0.666	0.490	0.718	0.431	0.743
**RNAalifold (ClustalW)**	0.452	0.580	0.400	0.662	0.333	0.725
**RNAalifold (MAFFT)**	0.614	0.704	0.565	0.751	0.510	**0.776**
**TurboFold**	0.780	0.750	0.808	**0.762**	0.815	0.762

	SRP					
	5 sequences		10 sequences		20 sequences	
	sensitivity	PPV	sensitivity	PPV	sensitivity	PPV
**TurboFold II**	**0.752**	0.823	**0.762**	0.828	**0.767**	0.831
**LocARNA**	0.713	0.732	0.674	0.748	0.616	0.772
**MaxExpect**	0.665	0.682	0.654	0.671	0.657	0.674
**MXSCARNA**	0.707	0.703	0.714	0.722	0.725	0.736
**RNAalifold (Clustal Omega)**	0.192	0.671	0.125	**0.887**	0.110	**0.922**
**RNAalifold (ClustalW)**	0.273	0.552	0.147	0.797	0.103	0.908
**RNAalifold (MAFFT)**	0.490	0.771	0.383	0.863	0.320	0.894
**TurboFold**	0.736	**0.824**	0.751	0.832	0.760	0.838

	telomerase					
	5 sequences		10 sequences		20 sequences	
	sensitivity	PPV	sensitivity	PPV	sensitivity	PPV
**TurboFold II**	**0.825**	0.679	**0.833**	0.683	**0.835**	0.681
**LocARNA**	0.616	0.510	0.611	0.571	0.581	0.615
**MaxExpect**	0.581	0.433	0.582	0.434	0.583	0.434
**MXSCARNA**	0.652	0.538	0.703	0.608	0.712	0.625
**RNAalifold (Clustal Omega)**	0.489	0.539	0.357	0.662	0.268	**0.811**
**RNAalifold (ClustalW)**	0.447	0.452	0.300	0.555	0.223	0.721
**RNAalifold (MAFFT)**	0.599	0.584	0.468	0.671	0.368	0.776
**TurboFold**	0.824	**0.683**	0.831	**0.684**	0.832	0.681

Section 6. Performance of alignment methods on BRAliBase 2.0

The benchmark of TurboFold II on BRAliBase 2.0 (11) is provided here. BraliBase 2.0 includes segments of group II introns, rRNA, SRP RNA, tRNA, and U5 RNA. The Sum of Pairs Score (SPS) is the ratio between the total number of columns in which nucleotide pairs or nucleotides aligned to a gap are identically aligned in dataset. The benchmark results of other methods are from the BRAliBase website: http://projects.binf.ku.dk/pgardner/bralibase/bralibase2.html.

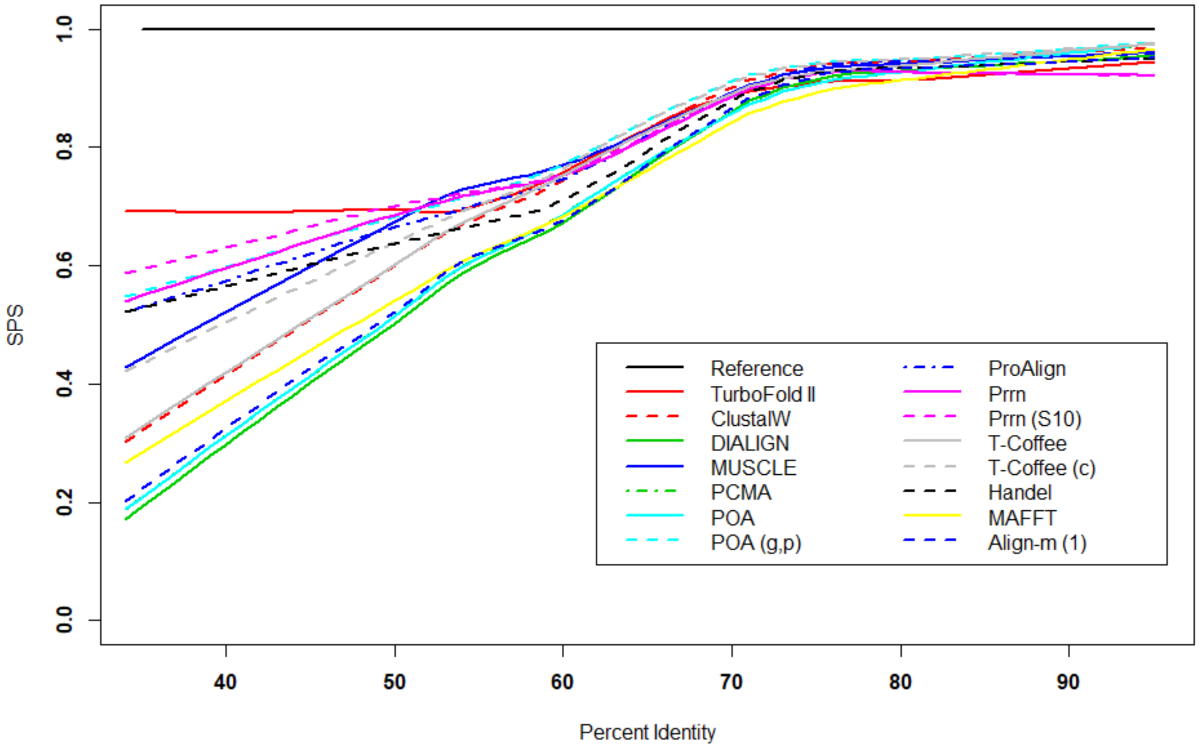

Figure S3. **The plot shows the alignment accuracy as function of the sequence identity.** The SPSs for methods other than TurboFold II are obtained from the supplementary data for BRAliBase 2.0 (11). The trend lines are plotted using LOWESS (locally weighted scatterplot smoothing) in R with the smoother span set to 0.5.

Section 6. Known and predicted structures and alignments for RNase P RNA from *Nocardioides albus*, *Propioniferax innocua*, *Salt Marsh A26, Mycobacterium tuberculosis, and Lake Griffy A #8*.

Figure S4 show the database structures for *Nocardioides albus*, *Propioniferax innocua*, *Salt Marsh A26*, *Mycobacterium tuberculosis,* and *Lake Griffy A #8* from the RNase P database (6). Figure S5 shows the database alignments for *Nocardioides albus*, *Propioniferax innocua*, *Salt Marsh A26*, *Mycobacterium tuberculosis* and *Lake Griffy A #8*. Figure S6-S11 show the predicted sequence alignments by TurboFold II, Clustal Omega, ClustalW, LocARNA and MXSCARNA and ProbCons, respectively, for this set of H=5 sequences.

Figure S12-S18 show the predicted structures by TurboFold II, LocARNA, MaxExpect, MXSCARNA, RNAalifold (using Clustal Omega alignment), RNAalifold (using ClustalW alignment), and TurboFold, respectively, for this set of H=5 sequences. For LocARNA, RNAalifold, and MXSCARNA, which predict consensus structure for multiple sequences, to avoid the inconsistency between the consensus structure and each sequence, the predicted structures for each sequence were generated by removing base pairing between nucleotide and insertion/deletion (gap) positions.

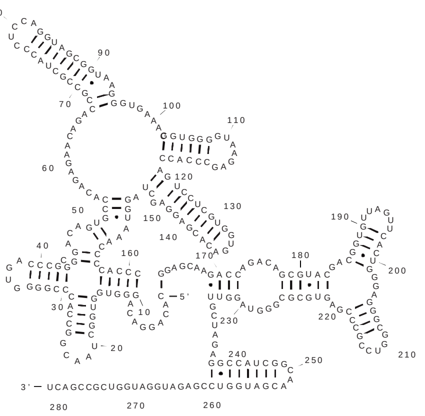

(a) *Nocardioides albus*

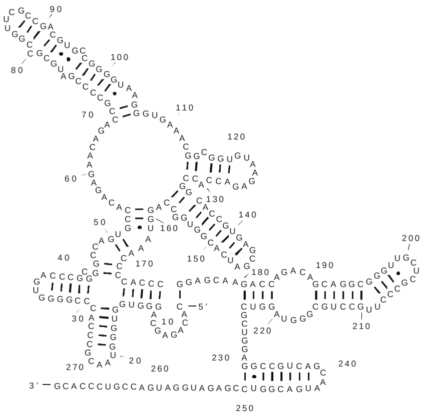

(b) *Propioniferax innocua*

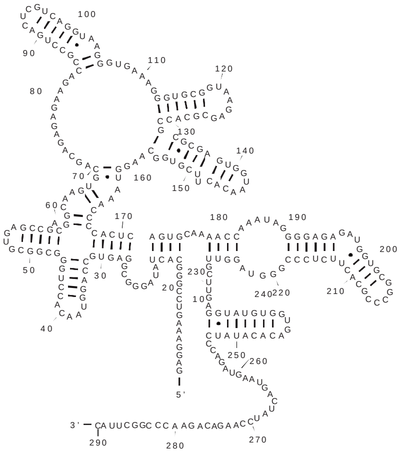

(c) *Salt Marsh A26*

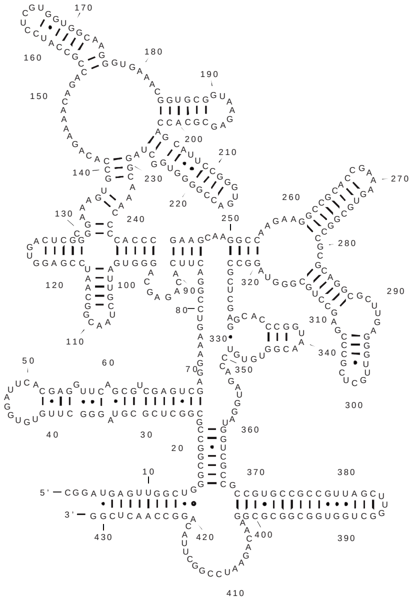

(d) *Mycobacterium tuberculosis*

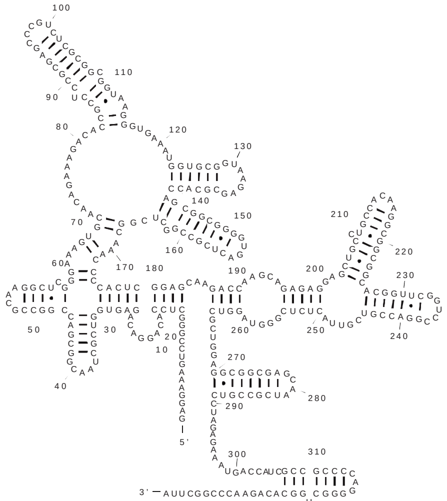

(e) *Lake Griffy A #8*

Figure S4. **Known structures for *Nocardioides albus*, *Propioniferax innocua*, *Salt Marsh A26*, *Mycobacterium tuberculosis* and *Lake Griffy A #8*.** Known structures for *Nocardioides albus* (a),

Propioniferax innocua (b), *Salt Marsh A26* (c), *Mycobacterium tuberculosis* (d) and *Lake Griffy A #8* (e) from the RNase P database (6). 5' and 3' ends of sequences are indicated by "5'" and "3'". A heavy line between two nucleotides indicates the base pairing interaction between the nucleotides.

```
N.albus             ------------------------------------------------------------
SM-A26              ------------------------------------------------------------
M.tuberculosis      CGGAUGAGUUGGCUGGGCGGCCGCGGCUCGCGUAGGGCUUGUGUGGAUUCACGAGGUUCA
LGA8                ------------------------------------------------------------
P.innocua           ------------------------------------------------------------

N.albus             ---------------------------CACAGGACAGGGUGGUGGCUAACGGCCACCCGG
SM-A26              ----------gaggaaagucCGGGCACUAUAGGGCGGAGUGCCAGGUAACACCUGGGCGG
M.tuberculosis      GCGUCGAGUCGAGGAAAGUCCGGACUUCACAGAGCAGGGUGAUUGCUAACGGCAAUCCGA
LGA8                ----------gaggaaaguccgggcUCCACAGGACAGAGUGGUCGCUAACGGCGACCGGC
P.innocua           ---------------------------CACAGAGCAGGGUGGUGGGUAACGCCCACCCGG

N.albus             GGUGACCCGCGGGACAGUGCCACAGAGAACAGA-CCGCCGCUACC------CUCCA----
SM-A26              CGUGAGCCGACGGCAAGUGCAGCAGAGAGAAG-ACCGCCUGA----------CUCG----
M.tuberculosis      GGUGACUCGCGGGAAAGUGCCACAGAAAACAG-ACCGCCAUC---------CUCGU----
LGA8                CGCAAGGCUCGGGAAAGUGCAACAGAAAGAC--ACCGCCUCCGCGAG----CCCGU----
P.innocua           GGUGACCCGCGGGCCAGUGCCACAGAGAACAGA-CCGCCCCGAUGCGCCGG-UUCGCCGA

N.albus             --GGUAGCGGUAAGGGUGAAACGGUGGGGUAAGAGCCCACCAGUCCUCGUGGUGACAC-G
SM-A26              -----UCAGGUAAGGGUGAAAGGGUGCGGUAAGAGCGCACC-GCGCGAGUGGUAACACUU
M.tuberculosis      -----GGUGGCAAGGGUGAAACGGUGCGGUAAGAGCGCACCAGCAUUCCGGGUGACCG-G
LGA8                CUCGCGGCGGUAAGGGUGAAAUGGUGCGGUAAGAGCGCACCAGCGGCGGG-GUGA-CU-C
P.innocua           CGUGCCGGGGUAAGGGUGAAACGGCGGUGUAAGAGACCACCGGCACCGUGAGCGAUCA-C

N.albus             AGGAGCU-AGGUAAACCCCACCCGGAGCAAGACCAGAC----------------------
SM-A26              CGUGGC-AAGGUAAACCCCACUCAGUGCAAAACCAAAU----------------------
M.tuberculosis      GGUGGCU-AGGCAAACCCCACCCGAAGCAAGGCCAAGAAGGCCGCACCGAAAGUGCGGCC
LGA8                GCCGGCU-CGGCAAACCCCACUCGGAGCAAGACCAAG-----------------------
P.innocua           GGUGGCC-AGGUAAACCCCACCCGGAGCAAGACCAGAC----------------------

N.albus             -AGCGUACGACGGUG-----UUAGUU----CACUGGG--AGGGCGGUCCGCCCGA-----
SM-A26              AGGGGAGAGA--------------------------U--GGUGCGGCCCGCACU------
M.tuberculosis      GCGCAGGCGC-------------------------UU-GAGGGUUGCUCGCCCGA-----
LGA8                CAGAGAG-GAGCUGCCUGCC-ACAA-GGCGGCGGC--ACGGUUCGGUCCGGACCGUCGUU
P.innocua           -AGCAGGC--------------------------------GGGUUGCUCGCCC------U

N.albus             -GUGCGCGGGUAGGUUGCUAGAGGCCAUCGGCAACGAUGGUCCGAGAUGGAUGGUCGCCG
SM-A26              -UCUCCCGGGUAGGUUGCUUGAGGUAUGUGGUGACACAUAUCCCAGAUGAAUGACUAUCC
M.tuberculosis      -GCCUGCGGGUAGGCCGCUCGAGGCACCCGGUAACGGUGUGUCCAGAUGGAUGGUCGCC-
LGA8                A-CUCUCGGGUAGGUCGCUGGAGGCGGCGAGCAAUCGCCGUCCUAGAGAAAUGACCAUC-
P.innocua           UGCCUGCGGGUAGGUCGCUGGAGGCCGUCAGCAAUGACGGUCCGAGAUGGAUGACCGUCC

N.albus             A----------------------------------------CU-----------------
SM-A26              A----------------------------------------AGacagaacccggcuuac-
M.tuberculosis      --GCCGUGCCGCCGUUAGCUUG-GCUgGUGGCGGCGC-GGA--ACAGAAUCCGGCUUACA
LGA8                --GCCGCCC-----------CAG-----------GGCUGGC--acagaacccggcuua--
P.innocua           CA---------------------------------------CG-----------------

N.albus             --------------
SM-A26              --------------
M.tuberculosis      GGCCAACUCGUCCG
LGA8                --------------
P.innocua           --------------
```

Figure S5. **Database alignments for *Nocardioides albus*, *Propioniferax innocua*, *Salt Marsh A26*, *Mycobacterium tuberculosis* and *Lake Griffy A #8*.**

```
N.albus           ------------------------------------------------------------
SM-A26(42)        -GA---------------------------------------------------------
M.tuberculosis    CGGAUGAGUUGGCUGGGCGGCCGCGGCUCGCGUAGGGCUUGUGUGGAUUCACGAGGUUCA
LGA8              GA----------------------------------------------------------
P.innocua         ------------------------------------------------------------

N.albus           ----------------------------CACAGGACAGGGUGGUGGCUAACGGCCACCCG
SM-A26(42)        ------------GGAAAGUCCGGGCACUAU-AGGGCGGAGUGCCAGGUAACACCUGGGCG
M.tuberculosis    GCGUCGAGUCGAGGAAAGUCCGGACUUCAC-AGAGCAGGGUGAUUGCUAACGGCAAUCCG
LGA8              ------------GGAAAGUCCGGGCUCCAC-AGGACAGAGUGGUCGCUAACGGCGACCGG
P.innocua         --------------------------CAC--AGAGCAGGGUGGUGGGUAACGCCCACCCG

N.albus           GGGUGACCCGCGGGACAGUGCCACAGAGAACAGACCGCCGCUAC------CCUCCA----
SM-A26(42)        GCGUGAGCCGACGGCAAGUGCAGCAGAGAGAAGACCGCCUG---------ACUCGU----
M.tuberculosis    AGGUGACUCGCGGGAAAGUGCCACAGAAAACAGACCGCCAU---------CCUCGU----
LGA8              CCGCAAGGCUCGGGAAAGUGCAACAGAAA-GACACCGCCUCCGC----GAGCCCGU---C
P.innocua         GGGUGACCCGCGGGCCAGUGCCACAGAGAACAGACCGCCCCGAUGCGCCGGUUCGCCGAC

N.albus           -GGUAGCGGUAAGGGUGAAACGGUGGGGUAAGAGCCCACCAGUCCUCGUGGUGACAC-GA
SM-A26(42)        -----CAGGUAAGGGUGAAAGGGUGCGGUAAGAGCGCACCGCGCG-AGUGGUAACACUUC
M.tuberculosis    ----GGUGGCAAGGGUGAAACGGUGCGGUAAGAGCGCACCAGCAUUCCGGGUGACCG-GG
LGA8              UCGCGGCGGUAAGGGUGAAAUGGUGCGGUAAGAGCGCACCAGCGG-CGGGGUGACUC--G
P.innocua         GUGCCGGGGUAAGGGUGAAACGGCGGUGUAAGAGACCACCGGCACCGUGAGCGAUCA-CG

N.albus           GGAGCUAGGUAAACCCCACCCGGAGCAAGACCAGACAGCGUACGACG----GUGUUAGUU
SM-A26(42)        GUGGCAAGGUAAACCCCACUCAGUGCAAAACCAAAUAGGGGAGAG--------------
M.tuberculosis    GUGGCUAGGCAAACCCCACCCGAAGCAAGGCCAAGAAGGCCGCA-------CCGAAAGUG
LGA8              CCGGCUCGGCAAACCCCACUCGGAGCAAGACCAAGCAGAGAGGAGCUGCCUGCCACAAGG
P.innocua         GUGGCCAGGUAAACCCCACCCGGAGCAAGACCAGACAGCAGGC-----------------

N.albus           CACUGGGA-----------GGGCGGUCCGCCC-----GAGUGCGCGGGUAGGUUGCUAGA
SM-A26(42)        -------AUG---------GUGCGGCCCGCAC-----U-UCUCCCGGGUAGGUUGCUUGA
M.tuberculosis    CGGCCGCGCAGGCGCUUGAGGGUUGCUCGCCC-----GAGCCUGCGGGUAGGCCGCUCGA
LGA8              CGGCGGCACG---------GUUCGGUCCGGACCGUCGUUACUCUCGGGUAGGUCGCUGGA
P.innocua         -------------------GGGUUGCUCGCCC-----UUGCCUGCGGGUAGGUCGCUGGA

N.albus           GGCCAUCGGCAACGAUGGUCCGAGAUGGAUGGUCGCCG----------------------
SM-A26(42)        GGUAUGUGGUGACACAUAUCCCAGAUGAAUGACUAUCC----------------------
M.tuberculosis    GGCACCCGGUAACGGUGUGUCCAGAUGGAUGGUCGCCGCCGUGCCGCCGUUAGCUUGGCU
LGA8              GGCGGCGAGCAAUCGCCGUCCUAGAGAAAUGACCAUCGCCGCCCC----------AG---
P.innocua         GGCCGUCAGCAAUGACGGUCCGAGAUGGAUGACCGUCC----------------------

N.albus           ----------------A-------------------------CU
SM-A26(42)        -----------AAGACAGAACCCGGCUU--------------AC
M.tuberculosis    GGUGGCGGCGCGGAACAGAAUCCGGCUUACAGGCCAACUCGUCCG
LGA8              ------GGGCUGGCACAGAACCCGGCUU----------------A
P.innocua         ---------------CA-------------------------CG
```

Figure S6. **Predicted alignments by TurboFold II for *Nocardioides albus*, *Propioniferax innocua*, *Salt Marsh A26*, *Mycobacterium tuberculosis* and *Lake Griffy A #8*.**

```
N.albus            ------------------------------------------------------------
SM-A26             ------------------------------------------------------------
M.tuberculosis     CGGAUGAGUUGGCUGGGCGGCCGCGGCUCGCGUAGGGCUUGUGUGGAUUCACGAGGUUCA
LGA8               ------------------------------------------------------------
P.innocua          ------------------------------------------------------------

N.albus            ---------------------------CACAGGACAGGGUGGUGGCUAACGGCCACCCGG
SM-A26             ----------GAGGAAAGUCCGGGCACUAUAGGGCGGAGUGCCAGGUAACACCUGGGCGG
M.tuberculosis     GCGUCGAGUCGAGGAAAGUCCGGACUUCACAGAGCAGGGUGAUUGCUAACGGCAAUCCGA
LGA8               ----------GAGGAAAGUCCGGGCUCCACAGGACAGAGUGGUCGCUAACGGCGACCGGC
P.innocua          ---------------------------CACAGAGCAGGGUGGUGGGUAACGCCCACCCGG

N.albus            GGUGACCCGCGGGACAGUGCCACAGAGAACAGACCGCCGCUACCCUCCAGGU--------
SM-A26             CGUGAGCCGACGGCAAGUGCAGCAGAGAGAAGACCGCCUGACUC---G------------
M.tuberculosis     GGUGACUCGCGGGAAAGUGCCACAGAAAACAGACCGCC---AUCCUCGU-----------
LGA8               CGCAAGGCUCGGGAAAGUGCAACAGAAA-GACACCGCCUCCGC----G---AGCCCGUCU
P.innocua          GGUGACCCGCGGGCCAGUGCCACAGAGAACAGACCGCCCCGAUGCGCCGGUUCGCCGACG

N.albus            ---AGCGGUAAGGGUGAAACGGUGGGGUAAGAGCCCACCAGUCCUCGUGGUGACAC-GAG
SM-A26             ---UCAGGUAAGGGUGAAAGGGUGCGGUAAGAGCGCACCGCGCGAG-UGGUAACACUUCG
M.tuberculosis     ---GGUGGCAAGGGUGAAACGGUGCGGUAAGAGCGCACCAGCAUUCCGGGUGACCG-GGG
LGA8               CGCGGCGGUAAGGGUGAAAUGGUGCGGUAAGAGCGCACCAGCGGCG-GGGUGACUC--GC
P.innocua          UGCCGGGGUAAGGGUGAAACGGCGGUGUAAGAGACCACCGGCACCGUGAGCGAUCA-CGG

N.albus            GAGCUAGGUAAACCCCACCCGGAGCAAGACCAGACAGCGUAC----------GACGGUGU
SM-A26             UGGCAAGGUAAACCCCACUCAGUGCAAAACCAAAUAGGGGAGAGAUG-------------
M.tuberculosis     UGGCUAGGCAAACCCCACCCGAAGCAAGGCCAAGAAGGCCGCACC-------GAAAGUGC
LGA8               CGGCUCGGCAAACCCCACUCGGAGCAAGACCAAGCAGAGAGGAGCUGCCUGCCACAAGGC
P.innocua          UGGCCAGGUAAACCCCACCCGGAGCAAGACCAGACAGCAGGC----------G-------

N.albus            UAGUUCA-----CUGGGAGGGCGGUCCGCCCGAGUGCGCGGGUAGGUUGCUAGAGGCCAU
SM-A26             ------------------GUGCGGCCCGCACUU-CUCCCGGGUAGGUUGCUUGAGGUAUG
M.tuberculosis     GGCCGCGCAGGCGCUUGAGGGUUGCUCGCCCGAGCCUGCGGGUAGGCCGCUCGAGGCACC
LGA8               GGCGGCACGG----UUCGGUCCGGACCGUCGUUACUCUCGGGUAGGUCGCUGGAGGCGGC
P.innocua          -------------------GGUUGCUCGCCCUUGCCUGCGGGUAGGUCGCUGGAGGCCGU

N.albus            CGGCAACGAUGGUCCGAGAUGGAUGGUCGCCGACU-------------------------
SM-A26             UGGUGACACAUAUCCCAGAUGAAUGACUAUCCA---------------------------
M.tuberculosis     CGGUAACGGUGUGUCCAGAUGGAUGGUCGCCGCCGUGCCGCCGUUAGCUUGGCUGGUGGC
LGA8               GAGCAAUCGCCGUCCUAGAGAAAUGACCAUCGCCGCCCCAG-------------------
P.innocua          CAGCAAUGACGGUCCGAGAUGGAUGACCGUCCCACG------------------------

N.albus            ---------------------------------------
SM-A26             ------AGACAGAACCCGGCUUAC---------------
M.tuberculosis     GGCGCGGAACAGAAUCCGGCUUACAGGCCAACUCGUCCG
LGA8               GGGCUGGCACAGAACCCGGCUUA----------------
P.innocua          ---------------------------------------
```

Figure S7. **Predicted alignments by Clustal Omega for *Nocardioides albus*, *Propioniferax innocua*, *Salt Marsh A26*, *Mycobacterium tuberculosis* and *Lake Griffy A #8*.**

```
N.albus          ------------------------------------------------------------
M.tuberculosis   CGGAUGAGUUGGCUGGGCGGCCGCGGCUCGCGUAGGGCUUGUGUGGAUUCACGAGGUUCA
P.innocua        ------------------------------------------------------------
SM-A26           ------------------------------------------------------------
LGA8             ------------------------------------------------------------

N.albus          ---------------------------CACAGGACAGGGUGGUGGCUAACGGCCACCCGG
M.tuberculosis   GCGUCGAGUCGAGGAAAGUCCGGACUUCACAGAGCAGGGUGAUUGCUAACGGCAAUCCGA
P.innocua        ---------------------------CACAGAGCAGGGUGGUGGGUAACGCCCACCCGG
SM-A26           ----------GAGGAAAGUCCGGGCACUAUAGGGCGGAGUGCCAGGUAACACCUGGGCGG
LGA8             ----------GAGGAAAGUCCGGGCUCCACAGGACAGAGUGGUCGCUAACGGCGACCGGC

N.albus          GGUGACCCGCGGGACAGUGCCACAGAGAACAGACCGCCGCUACCCUCCAG----------
M.tuberculosis   GGUGACUCGCGGGAAAGUGCCACAGAAAACAGACCGCC---AUCCUC-------------
P.innocua        GGUGACCCGCGGGCCAGUGCCACAGAGAACAGACCGCCCCGAUGCGCCGGUUCGCCGACG
SM-A26           CGUGAGCCGACGGCAAGUGCAGCAGAGAGAAGACCGCCU-----GACUCGUCA-------
LGA8             CGCAAGGCUCGGGAAAGUGCAACAGAAAGAC-ACCGCCUCCGCGAGCCCGUCUC------

N.albus          -GUAGCGGUAAGGGUGAAACGGUGGGGUAAGAGCCCACCAGUCCUCGUGGUGACACGAGG
M.tuberculosis   -GUGGUGGCAAGGGUGAAACGGUGCGGUAAGAGCGCACCAGCAUUCCGGGUGACCGGGGU
P.innocua        UGCCGGGGUAAGGGUGAAACGGCGGUGUAAGAGACCACCGGCACCGUGAGCGAUCACGGU
SM-A26           ------GGUAAGGGUGAAAGGGUGCGGUAAGAGCGCACCGCGCGAGUGGUAACACUUCGU
LGA8             -GCGGCGGUAAGGGUGAAAUGGUGCGGUAAGAGCGCACCAGCGGCGGGGUGACUCGCCG-

N.albus          AGCUAGGUAAACCCCACCCGGAGCAAGACCAGACAG--CGUAC-GACGGUGUUAGUUCAC
M.tuberculosis   GGCUAGGCAAACCCCACCCGAAGCAAGGCCAAGAAGGCCGCACCGAAAGUGCGGCCGCGC
P.innocua        GGCCAGGUAAACCCCACCCGGAGCAAGACCAGACAG-----------------------C
SM-A26           GGCAAGGUAAACCCCACUCAGUGCAAAACCAAAUAGG--GGAGAG---------------
LGA8             -GCUCGGCAAACCCCACUCGGAGCAAGACCAAGCAGA--GAGGAGCUGCCUGCCACAAGG

N.albus          UGG-----GAGGGCGGUCCGCCC-----GAGUGCGCGGGUAGGUUGCUAGAGGCCAUCGG
M.tuberculosis   AGGCGCUUGAGGGUUGCUCGCCC-----GAGCCUGCGGGUAGGCCGCUCGAGGCACCCGG
P.innocua        AGGC------GGGUUGCUCGCCC-----UUGCCUGCGGGUAGGUCGCUGGAGGCCGUCAG
SM-A26           -------AUGGUGCGGCCCGCAC------UUCUCCCGGGUAGGUUGCUUGAGGUAUGUGG
LGA8             CGGCGGCACGGUUCGGUCCGGACCGUCGUUACUCUCGGGUAGGUCGCUGGAGGCGGCGAG

N.albus          CAACGAUGGUCCGAGAUGGAUGGUCGCCGACU----------------------------
M.tuberculosis   UAACGGUGUGUCCAGAUGGAUGGUCGCCGCCGUGCCGCCGUUAGCUUGGCUGGUGGCGGC
P.innocua        CAAUGACGGUCCGAGAUGGAUGACCGUC-CCACG--------------------------
SM-A26           UGACACAUAUCCCAGAUGAAUGACUAUC-------CAAG-------ACAGAACCCGGCUU
LGA8             CAAUCGCCGUCCUAGAGAAAUGACCAUCGCCGCCCCAGGGGCUGGCACAGAACCCGGCUU

N.albus          ------------------------------------
M.tuberculosis   GCGGAACAGAAUCCGGCUUACAGGCCAACUCGUCCG
P.innocua        ------------------------------------
SM-A26           AC----------------------------------
LGA8             A-----------------------------------
```

Figure S8. **Predicted alignments by ClustalW for *Nocardioides albus*, *Propioniferax innocua*, *Salt Marsh A26*, *Mycobacterium tuberculosis* and *Lake Griffy A #8*.**

```
SM-A26            GAGGAAAGUCCGGGCACUAUAG--GGCGGAGU--GCCAGG--------UAACACCUGGGC
LGA8              GAGGAAAGUCCGGGCUCCACAG--GACAGAGU--GGUCGC--------UAACGGCGACCG
N.albus           -----------------CACAG--GACAGGGU--GGUGGC--------UAACGGCCACCC
P.innocua         -----------------CACAG--AGCAGGGU--GGUGGG--------UAACGCCCACCC
M.tuberculosis    CGGAUGAGUUGGCUGGGCGGCCGCGGCUCGCGUAGGGCUUGUGUGGAUUCACGAGGUUCA

SM-A26            GGCGUGAGCC---GACGGCAAGUGCAGCAGAGAG-AA-------GAC----CGCCUGAC-
LGA8              GCCGCAAGGC---UCGGGAAAGUGCAACAGAAAG-AC-------ACCGCCUCCGCGAGC-
N.albus           GGGGUGACCC---GCGGGACAGUGCCACAGAGAACAG-------ACCGCCGCUACCCUC-
P.innocua         GGGGUGACCC---GCGGGCCAGUGCCACAGAGAACAG-------ACCGCCCCGAUGCGCC
M.tuberculosis    GCGUCGAGUCGAGGAAAGUCCGGACUUCACAGAGCAGGGUGAUUGCUAACGGCAAUCCGA

SM-A26            ------UCG-UCA---GGU----------------------------------AAGGGUGA
LGA8              ------CCGUCUC---GCGGC----------------------------GGUAAGGGUGA
N.albus           ----------CAG---GUAGC----------------------------GGUAAGGGUGA
P.innocua         GGUUCGCCGACGU---GCCGG----------------------------GGUAAGGGUGA
M.tuberculosis    GGUGACUCGCGGGAAAGUGCCACAGAAAACAGACCGCCAUCCUCGUGGUGGCAAGGGUGA

SM-A26            AAGGGUGCGGUAAGAGCGCACCGCGCG-AGUGGUAACACUUCGUGGCAAGGUAAACCCCA
LGA8              AAUGGUGCGGUAAGAGCGCACCAGCGG-CGGGGUGACUCG-CCGGCU-CGGCAAACCCCA
N.albus           AACGGUGGGGUAAGAGCCCACCAGUCCUCGUGGUGACACGAGGAGCU-AGGUAAACCCCA
P.innocua         AACGGCGGUGUAAGAGACCACCGGCACCGUGAGCGAUCACGGUGGCC-AGGUAAACCCCA
M.tuberculosis    AACGGUGCGGUAAGAGCGCACCAGCAUUCCGGGUGACCGGGGUGGCU-AGGCAAACCCCA

SM-A26            CUCAGUGCAAAACCAAAUAGGGGAGAG----------------------AUGGUGCGGCC
LGA8              CUCGGAGCAAGACCAAGCAGAGAGGAGCUGCCUGCCACAAGGCGGCGGCACGGUUCGGUC
N.albus           CCCGGAGCAAGACCAGACAGCGUACGACGGUGUU-----AGUUCACUG-GGAGGGCGGUC
P.innocua         CCCGGAGCAAGACCAGACAGCAGGC--------------------------GGGUUGCU
M.tuberculosis    CCCGAAGCAAGGCCAAGAAGGCCGCACCGAAAGUGCGGCCGCGCAGGC-GCUUGAGGGUU

SM-A26            CGCACU-----UCUCCC-GGGUAGGUUGCUUGAGGUAUGUGGUGACAC-AUAUCCCAGAU
LGA8              CGGACCGUCGUUACUCUCGGGUAGGUCGCUGGAGGCGGCGAGCAAUCG-CCGUCCUAGAG
N.albus           CGCCCG-----AGUGCGCGGGUAGGUUGCUAGAGGCCAUCGGCAACGA-UGGUCCGAGAU
P.innocua         CGCCCU-----UGCCUGCGGGUAGGUCGCUGGAGGCCGUCAGCAAUGA-CGGUCCGAGAU
M.tuberculosis    GCUCGCCCGAGCCUGC--GGGUAGGCCGCUCGAGGCACCCGGUAACGGUGUGUCC-AGAU

SM-A26            GAAUGACUAU----------------CCAAG-----------------ACAGAACCCGGC
LGA8              AAAUGACCAUCGCC----------GCCCCAGGGGC---------UGGCACAGAACCCGGC
N.albus           GGAUGGUCG-------------------------------------------------CCGAC
P.innocua         GGAUGACCGU------------------------------------------------CCCAC
M.tuberculosis    GGAUGGUCGCCGCCGUGCCGCCGUUAGCUUGGCUGGUGGCGGCGCGGAACAGAAUCCGGC

SM-A26            UUAC---------------
LGA8              UUA----------------
N.albus           U------------------
P.innocua         G------------------
M.tuberculosis    UUACAGGCCAACUCGUCCG
```

Figure S9. **Predicted alignments by LocARNA for *Nocardioides albus*, *Propioniferax innocua*, *Salt Marsh A26*, *Mycobacterium tuberculosis* and *Lake Griffy A #8*.**

```
N.albus            CACA--------------------------------------------------------
SM-A26             GA----------------------------------------------------------
M.tuberculosis     CGGAUGAGUUGGCUGGGCGGCCGCGGCUCGCGUAGGGCUUGUGUGGAUUCACGAGGUUCA
LGA8               GA----------------------------------------------------------
P.innocua          C-----------------------------------------------------------

N.albus            -------------------------------GGACAGGGUGGUGGCUAACGGCCACCCGG
SM-A26             ------------GGAAAGUCCGGGCACUAUAGGGCGGAGUGCCAGGUAACACCUGGGCGG
M.tuberculosis     GCGUCGAGUCGAGGAAAGUCCGGACUUCACAGAGCAGGGUGAUUGCUAACGGCAAUCCGA
LGA8               ------------GGAAAGUCCGGGCUCCACAGGACAGAGUGGUCGCUAACGGCGACCGGC
P.innocua          ----------------------------ACAGAGCAGGGUGGUGGGUAACGCCCACCCGG

N.albus            GGUGACCCGCGGGACAGUGCCACAGAGAACAGACCGCCGCU---AC--C----C---UCC
SM-A26             CGUGAGCCGACGGCAAGUGCAGCAGAGAGAAGACCGCC------UGACU----C---GUC
M.tuberculosis     GGUGACUCGCGGGAAAGUGCCACAGAAAACAGACCGCC------AU--C----C---UCG
LGA8               CGCAAGGCUCGGGAAAGUGCAACAGAAAG-ACACCGCCUCCGCGAG-CC----C---GUC
P.innocua          GGUGACCCGCGGGCCAGUGCCACAGAGAACAGACCGCCCCGAUGCG-CCGGUUCGCCGAC

N.albus            AGGUAGCGGUAAGGGUGAAACGGUGGGGUAAGAGCCCACCAGUCCUCG-UGGUGAC-ACG
SM-A26             ------AGGUAAGGGUGAAAGGGUGCGGUAAGAGCGCACCGCGCG-AG-UGGUAAC-ACU
M.tuberculosis     UG---GUGGCAAGGGUGAAACGGUGCGGUAAGAGCGCACCAGCAUUCC-GGGUGAC-CGG
LGA8               UCGCGGCGGUAAGGGUGAAAUGGUGCGGUAAGAGCGCACCAGCGG-CG-GGGUGAC-UCG
P.innocua          GUGCCGGGGUAAGGGUGAAACGGCGGUGUAAGAGACCACCGGCAC-CGUGAGCGAUCACG

N.albus            -AGGAGCUAGGUAAACCCCACCCGGAGCAAGACCAGACAGC--GUA-CG---ACGGUGUU
SM-A26             UCGUGGCAAGGUAAACCCCACUCAGUGCAAAACCAAAUAGG--GGAGAG-----------
M.tuberculosis     -GGUGGCUAGGCAAACCCCACCCGAAGCAAGGCCAAGAAGGCCGCACCG---AAAGUGCG
LGA8               --CCGGCUCGGCAAACCCCACUCGGAGCAAGACCAAGCAGA--GAGGAGCUGCCUGCCAC
P.innocua          --GUGGCCAGGUAAACCCCACCCGGAGCAAGACCAGACAGC--AGGCGG-----------

N.albus            AGUUC--ACUGG-----GAGG--GCGGUCCG-----CCCGAGUGCGCGGGUAGGUUGCUA
SM-A26             ------------------AUGGUGCGGCCCGCA------CUUCUCCCGGGUAGGUUGCUU
M.tuberculosis     GCCGC--GCAGGCGCUUGAGG--GUUGCUCG-----CCCGAGCCUGCGGGUAGGCCGCUC
LGA8               A--AGGCGGCGG-----CACGGUUCGGUCCGGACCGUCGUUACUCUCGGGUAGGUCGCUG
P.innocua          ---------------------G--UUGCUCG-----CCCUUGCCUGCGGGUAGGUCGCUG

N.albus            GAGGCCAUCGGCAACGAUGGUCCGAGAUGGAUGGUCGC----------------------
SM-A26             GAGGUAUGUGGUGACACAUAUCCCAGAUGAAUGACUAUC-------C------A------
M.tuberculosis     GAGGCACCCGGUAACGGUGUGUCCAGAUGGAUGGUCGCCGCCGUGCCGCCGUUAGCUUGG
LGA8               GAGGCGGCGAGCAAUCGCCGUCCUAGAGAAAUGACCAUCGCCGCCCC------AG--GGG
P.innocua          GAGGCCGUCAGCAAUGACGGUCCGAGAUGGAUGACCGUC-------C-------------

N.albus            ---------------------------C-G--------------ACU
SM-A26             --------------AGACAGAACCCGGCU---------------UAC
M.tuberculosis     CUGGUGGCGGCGCGGAACAGAAUCCGGCUUACAGGCCAACUCGUCCG
LGA8               C-----------UGGCACAGAACCCGGCU---------------U-A
P.innocua          ----------------------------C---------------ACG
```

Figure S10. **Predicted alignments by MXSCARNA for *Nocardioides albus*, *Propioniferax innocua*, *Salt Marsh A26*, *Mycobacterium tuberculosis* and *Lake Griffy A #8*.**

```
N.albus           --------------------------------------------------------
SM-A26            --------------------------------------------------------
M.tuberculosis    CGGAUGAGUUGGCUGGGCGGCCGCGGCUCGCGUAGGGCUUGUGUGGAUUCACGAGG
LGA8              --------------------------------------------------------
P.innocua         --------------------------------------------------------

N.albus           ---------------------------CAC---AGGACAGGGUGGUGGCUAACGGC
SM-A26            ----------GAGGAAAGUCCGGG---CACUAUAGGGCGGAGUGCCAGGUAACACC
M.tuberculosis    GCGUCGAGUCGAGGAAAGUCCGGACUUCAC---AGAGCAGGGUGAUUGCUAACGGC
LGA8              ----------GAGGAAAGUCCGGGCUCCAC---AGGACAGAGUGGUCGCUAACGGC
P.innocua         ---------------------------CAC---AGAGCAGGGUGGUGGGUAACGCC

N.albus           CCGGGGUGACCCG-------CGGGACAGUGCCACAGAGAACAGACCGCC--G--CU
SM-A26            GCGGCGUGAGCCG-------ACGGCAAGUGCAGCAGAGAGAAGACCGCC-UG----
M.tuberculosis    CCGAGGUGACUCG-------CGGGAAAGUGCCACAGAAAACAGACCGCCAUCC---
LGA8              CCGG------CCGCAAGGCUCGGGAAAGUGCAACAGAAAG-ACACCGCC-UCCGC-
P.innocua         CCGGGGUGACCCG-------CGGGCCAGUGCCACAGAGAACAGACCGCC--CCGAU

N.albus           -C--CUCCAG----GUAG--CGGUAAGGGUGAAACGGUGGGG--UAAGAGCCCACC
SM-A26            ----CUCGUC----------AGGUAAGGGUGAAAGGGUGCGG--UAAGAGCGCACC
M.tuberculosis    -----UCGUG----GUGG--C---AAGGGUGAAACGGUGCGG--UAAGAGCGCACC
LGA8              CCGUCUCG-C------GG--CGGUAAGGGUGAAAUGGUGCGG--UAAGAGCGCACC
P.innocua         CGGU-UCG-CCGACGUGCCGGGGUAAGGGUGAAACG--GCGGUGUAAGAGACCACC

N.albus           CU--CG--UGGUGAC-A--CGA--GG-AGCUA-GGUAAACCCCACCCGGAGCAAGA
SM-A26            G---CGAGUGGUAAC-----ACUUCGUGGC-AAGGUAAACCCCACUCAGUGCAAAA
M.tuberculosis    AUUCCG---GGUGAC----CGG--GGUGGCUA-GGCAAACCCCACCCGAAGCAAGG
LGA8              G--GCGG--GGUGAC---UCGC--C--GGCUC-GGCAAACCCCACUCGGAGCAAGA
P.innocua         A--CCG-----UGAGCGAUCAC--GGUGGC-CAGGUAAACCCCACCCGGAGCAAGA

N.albus           A-CAGC--------------GUACGACGGUGUUAGUUCACUGGGAGGGCG------
SM-A26            AUAGGGG-A----GAGAUG-GUG-------------------------CG------
M.tuberculosis    G-AAGGCCGCACCGAAA---GUGCGGCC-------------GCGCAGGCGCUUGAG
LGA8              G-CAGAG----GAGCUGCCUGCCAC-----------------AAGGCG------
P.innocua         A-CAGC---------------------------------------AGGCG------

N.albus           -C-CGCCC----------GAG------------UGCGCGGGUAGGUUGCUAGAG--
SM-A26            ----GCCC----------GCAC-----U---UCU-CCCGGGUAGGUUGCUUGAGGU
M.tuberculosis    GCUCGCCC----------GAGC----------CU--GCGGGUAGGCCGCUCGAG--
LGA8              -C-GGCACGGUUCGGUCCGGACCGUCGUUACUCU---CGGGUAGGUCGCUGGAG--
P.innocua         GCUCGCCC--U------UGC------------CU--GCGGGUAGGUCGCUGGAG--

N.albus           ----GCCAUC-GGCA---ACGAUG-GUCC--GAGAUGGAUGG---UCG--------
SM-A26            GGUGAC------------ACA-U--AUCC--CAGAUGAAUGACUAUCC--------
M.tuberculosis    ----GCAC-C---CGGUAACGGUGUGUCC---AGAUGGAUGG---UCGCCGCCGUG
LGA8              ----GCGG-CGAGCAAUCGCC----GUCCUAGAGA--AAUGACCAUCGCCGCC---
P.innocua         ----GCCGUC-AGCAAUGACG----GUCC--GAGAUGGAUGACCGUCC--------

N.albus           -----------------------------------CCGACU---------------
SM-A26            --------------------AAGACAGAAC-----CCGGCUUAC------------
M.tuberculosis    CGUUAGCUUGGCUGGUGGC-GGCGCGGAACAGAAUCCGGCUUACAGGCCAACUCGU
LGA8              -------------GG-GGCUGGCACAGAAC-----CCGGCUUA-------------
P.innocua         -----------------------------------ACG------------------
```

Figure S11. **Predicted alignments by ProbCons for *Nocardioides albus*, *Propioniferax innocua*, *Salt Marsh A26*, *Mycobacterium tuberculosis* and *Lake Griffy A #8*.**

```
N.albus           CACA-----------------------------------------------------
SM-A26(42)        GAGGAAAGU---CCGGGCACU------------------------------------
M.tuberculosis    CGGAUGAGUUGGCUGGGCGGCCGCGGCUCGCGUAGGGCUUGUGUGGAUUCACGAGGU
LGA8              GAGGAAAGU---CCGGGCUCC------------------------------------
P.innocua         CACA-----------------------------------------------------

N.albus           -------------------------------GGACAGGGUGGUGGCUAACGGCCACC
SM-A26(42)        ----------------------------AUAGGGCGGAGUGCCAGGUAACACCUGGG
M.tuberculosis    GCGUCGAGUCGAGGAAAGUCCGGACUUCACAGAGCAGGGUGAUUGCUAACGGCAAUC
LGA8              ----------------------------ACAGGACAGAGUGGUCGCUAACGGCGACC
P.innocua         -------------------------------GAGCAGGGUGGUGGGUAACGCCCACC

N.albus           GGUGACCCGCGGGACAGUGCCACAGAGAACAGACCGCCGCUACCCUC----------
SM-A26(42)        CGUGAGCCGACGGCAAGUGCAGCAGAGAGAAGACCGCC-UGA---------------
M.tuberculosis    GGUGACUCGCGGGAAAGUGCCACAGAAAACAGACCGCC---AUCC------------
LGA8              CGCAAGGCUCGGGAAAGUGCAACAGAAAG-ACACCGCCUCCGCGAGCCCGU------
P.innocua         GGUGACCCGCGGGCCAGUGCCACAGAGAACAGACCGCCCCGAUGCGCCGGUUCGCCG

N.albus           GGUAGCGGUAAGGGUGAAACGGUGGGGUAAGAGCCCACCAGUCCUCGUGGUGACACG
SM-A26(42)        CGUCA-GGUAAGGGUGAAAGGGUGCGGUAAGAGCGCACC-GCGCGAGUGGUAACACU
M.tuberculosis    CGUGGUGGCAAGGGUGAAACGGUGCGGUAAGAGCGCACCAGCAUUCCGGGUGACCGG
LGA8              CGCGGCGGUAAGGGUGAAAUGGUGCGGUAAGAGCGCACCAGCG-GCGGGGUGACUCG
P.innocua         UGCCGGGGUAAGGGUGAAACGGCGGUGUAAGAGACCACCGGCACCGUGAGCGAUCAC

N.albus           GAGCUAGGUAAACCCCACCCGGAGCAAGACCAGACAGCGUACGACGG----UGUUAG
SM-A26(42)        UGGCAAGGUAAACCCCACUCAGUGCAAAACCAAAUAGGGGAG---AG----------
M.tuberculosis    UGGCUAGGCAAACCCCACCCGAAGCAAGGCCAAGAAGGCCGC---AC----CGAAAG
LGA8              CGGCUCGGCAAACCCCACUCGGAGCAAGACCAAGCAGAGAGGAGCUGCCUGCCACAA
P.innocua         UGGCCAGGUAAACCCCACCCGGAGCAAGACCAGACAGCAGGC---------------

N.albus           ACUGG-----------GAGGGCGGUCCGCCCGAG-----UGCGCGGGUAGGUUGCUA
SM-A26(42)        ------AUG---------GUGCGGCCCGCACU-U-----CUCCCGGGUAGGUUGCUU
M.tuberculosis    GGCCGCGCAGGCGCUUGAGGGUUGCUCGCCCGAG-----CCUGCGGGUAGGCCGCUC
LGA8              GGCGGCACG---------GUUCGGUCCGGACCGUCGUUACUCUCGGGUAGGUCGCUG
P.innocua         ------------------GGGUUGCUCGCCCUUG-----CCUGCGGGUAGGUCGCUG

N.albus           GCCAUCGGCAACGA-UGGUCCGAGAUGGAUGGUCG----------------------
SM-A26(42)        GUAUGUGGUGACAC-AUAUCCCAGAUGAAUGACU-----------------------
M.tuberculosis    GCACCCGGUAACGGUGUGUCC-AGAUGGAUGGUCGCCGCCGUGCCGCCGUUAGCUUG
LGA8              GCGGCGAGCAAUCG-CCGUCCUAGAGAAAUGACCAUC-----GCCGCCC-CAG----
P.innocua         GCCGUCAGCAAUGA-CGGUCCGAGAUGGAUGACC-----------------------

N.albus           ---------------------------------CCGA------CU
SM-A26(42)        ---AUC----CAAGACAGAAC------------CCGGCUU---AC
M.tuberculosis    GGUGGCGGCGCGGAACAGAAUCCGGCUUACAGGCCAACUCGUCCG
LGA8              --GGGCUG-GC---ACAGAAC------------CCGGCUU----A
P.innocua         ---GUC---------------------------CCA-------CG
```

Figure S12. **Predicted alignments by MAFFT for *Nocardioides albus*, *Propioniferax innocua*, *Salt Marsh A26*, *Mycobacterium tuberculosis* and *Lake Griffy A #8*.**

```
N.albus          -----------------CACA-GGAC------------------------------
SM-A26(42)       GAGGAAAGUCCGGGCACUAUA-GGGC------------------------------
M.tuberculosis   -----------------CGGAUGAGUUGGCUGGGCGGCCGCGGCUCGCGUAGGGCU
LGA8             GAGGAAAGUCCGGGCUCCACA-GGAC------------------------------
P.innocua        -----------------CACA-GAGC------------------------------

N.albus          ----------------------------------------------------AGGG
SM-A26(42)       ----------------------------------------------------GGAG
M.tuberculosis   UGGAUUCACGAGGUUCAGCGUCGAGUCGAGGAAAGUCCGGACUUCACAGAGCAGGG
LGA8             ----------------------------------------------------AGAG
P.innocua        ----------------------------------------------------AGGG

N.albus          GGCUAACGGCCACCCGGGGUGACCCGCGGGACAGUGCCACAGAGAACAG------A
SM-A26(42)       AGGUAACACCUGGGCGGCGUGAGCCGACGGCAAGUGCAGCAGAGAGAAG------A
M.tuberculosis   UGCUAACGGCAAUCCGAGGUGACUCGCGGGAAAGUGCCACAGAAAACAGACCGCCA
LGA8             CGCUAACGGCGACCGGCCGCAAGGCUCGGGAAAGUGCAACAGAAA-GAC------A
P.innocua        GGGUAACGCCCACCCGGGGUGACCCGCGGGCCAGUGCCACAGAGAACAGACC---G

N.albus          GCCGCUAC----CCU--------CC-A--GGUAGCGGUAAGGGUGAAACGGUGGGG
SM-A26(42)       GCC-UGAC----UC----------------GUC-AGGUAAGGGUGAAAGGGUGCGG
M.tuberculosis   -------------C------------U--CGUGGUGGCAAGGGUGAAACGGUGCGG
LGA8             GCCUCCGCGAGCCCG--------UC-U--CGCGGCGGUAAGGGUGAAAUGGUGCGG
P.innocua        -----GAU----GCGCCGGUUCGCCGACGUGCCGGGGUAAGGGUGAAACGGCGGUG

N.albus          AGCCCACCAGUCCUCGUGGUGACACG-AGGAGCUAGGUAAACCCCACCCGGAGCAA
SM-A26(42)       AGCGCACC-GCGCGAGUGGUAACACUUCGUGGCAAGGUAAACCCCACUCAGUGCAA
M.tuberculosis   AGCGCACCAGCAUUCCGGGUGACCGG-GGUGGCUAGGCAAACCCCACCCGAAGCAA
LGA8             AGCGCACCAGCGG-CGGGGUGACUCG--CCGGCUCGGCAAACCCCACUCGGAGCAA
P.innocua        AGACCACCGGCACCGUGAGCGAUCAC-GGUGGCCAGGUAAACCCCACCCGGAGCAA

N.albus          AGACAGC--GUA-CGACGGUGUUAG---UUCA-------CUGGGAGG-GCGGU---
SM-A26(42)       AAAUAGG--GGAGAGAUGG---------------------------U-GCGGC---
M.tuberculosis   AAGAAGGCCGCACCGAAAGUGCGGC---CGCGCAG-GCGCUU-GAGG-GUUGC---
LGA8             AAGCAGA--GAG-GA-----GCUGCCUGCC-ACAAGG----CGGCGGCACGGUUCG
P.innocua        AGACAGC--AGG-C-------------------------------GG-GUUGC---

N.albus          ----CGCCCGAGUGCGCGGGUAGGUUGCUAGAGGCCAUCGGCAACGA-UGGUCCGA
SM-A26(42)       ----CGCACUUCUCCC-GGGUAGGUUGCUUGAGGUAUGUGGUGACAC-AUAUCCCA
M.tuberculosis   ----CGCCCGAGCCUGCGGGUAGGCCGCUCGAGGCACCCGGUAACGGUGUGU-CCA
LGA8             GGACCGUCGUUACUCUCGGGUAGGUCGCUGGAGGCGGCGAGCAAUCG-CCGUCCUA
P.innocua        ----CGCCCUUGCCUGCGGGUAGGUCGCUGGAGGCCGUCAGCAAUGA-CGGUCCGA

N.albus          GAU--------------------------G----G---------------------
SM-A26(42)       AAU--------------------------GACU-AU------CC------------
M.tuberculosis   GAUGGUCGCCGCCGUGCCGCCGUUAGCUUGGCUGG------------UGGCGGCGC
LGA8             AAU--------------------------GACC-AUCGCCGCCCAGG-----GGC
P.innocua        GAU--------------------------G----A---------------------

N.albus          -------UCG-CCGA--------------CU
SM-A26(42)       ACAGAACCCGGCUU---------------AC
M.tuberculosis   ACAGAAUCCGGCUUACAGGCCAACUCGUCCG
LGA8             ACAGAACCCGGCU----------------UA
P.innocua        -------CCGUCCCA--------------CG
```

Figure S13. **Predicted alignments by R-Coffee for *Nocardioides albus*, *Propioniferax innocua*, *Salt Marsh A26*, *Mycobacterium tuberculosis* and *Lake Griffy A #8*.**

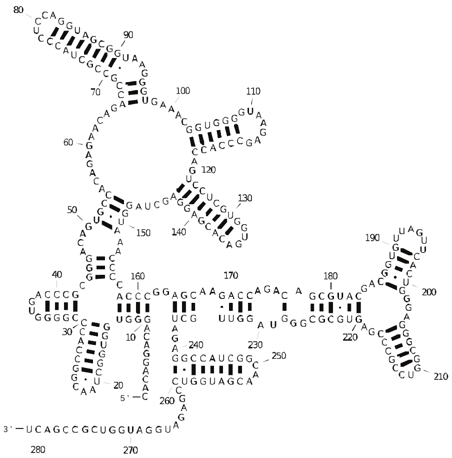

(a) *Nocardioides albus*

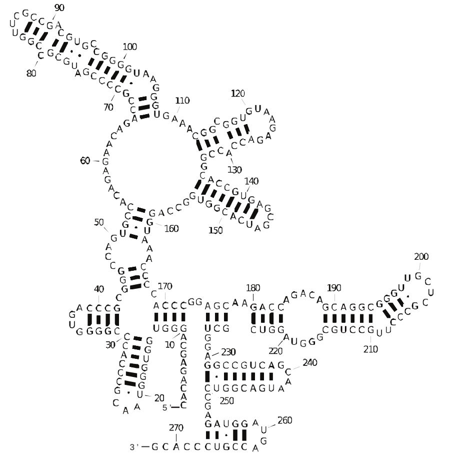

(b) *Propioniferax innocua*

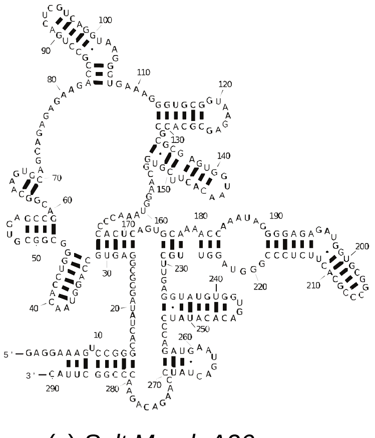

(c) *Salt Marsh A26*

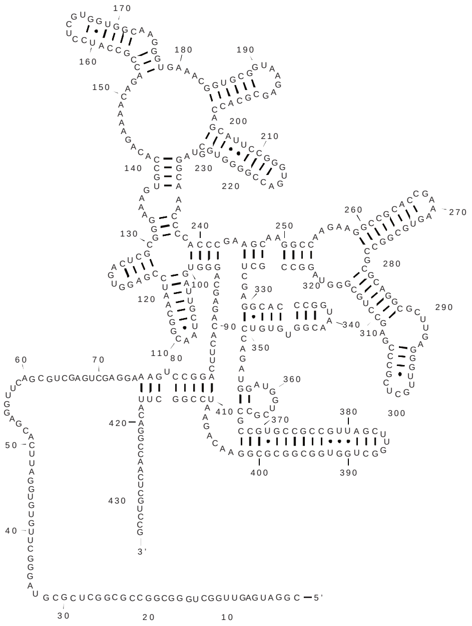

(d) *Mycobacterium tuberculosis*

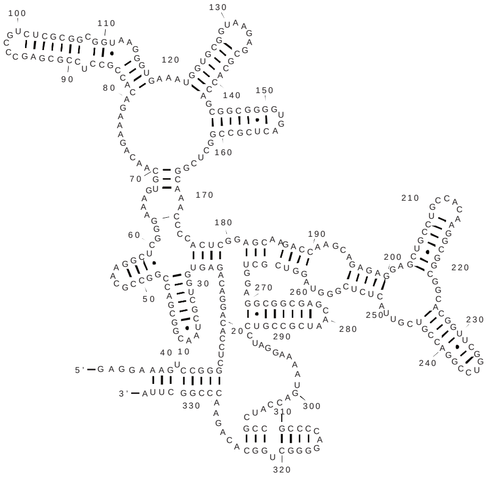

(e) *Lake Griffy A #8*

Figure S14. **Predicted structures by TurboFold II for *Nocardioides albus*, *Propioniferax innocua*, *Salt Marsh A26*, *Mycobacterium tuberculosis* and *Lake Griffy A #8*.** Predicted structures for *Nocardioides albus* (a), *Propioniferax innocua* (b), *Salt Marsh A26* (c), *Mycobacterium*

tuberculosis (d) and *Lake Griffy A #8* (e). 5’ and 3’ ends of sequences are indicated by “5’” and “3’”. A heavy line between two nucleotides indicates the base pairing interaction between the nucleotides.

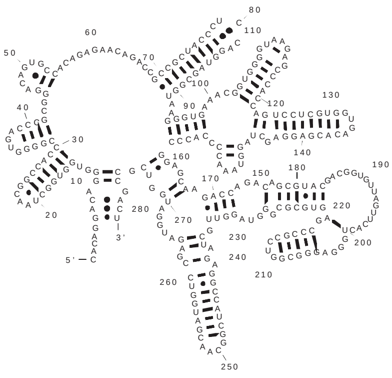

(a) *Nocardioides albus*

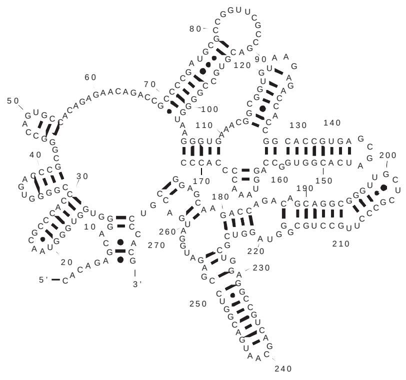

(b) *Propioniferax innocua*

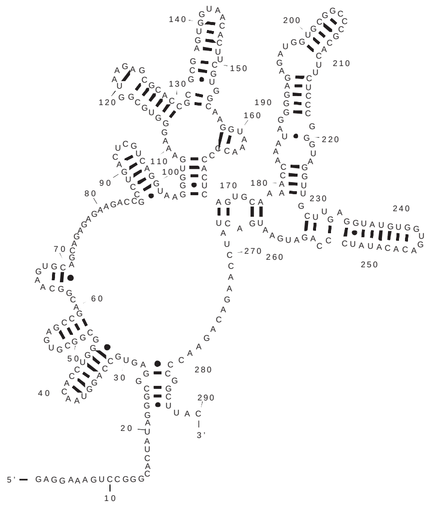

(c) *Salt Marsh A26*

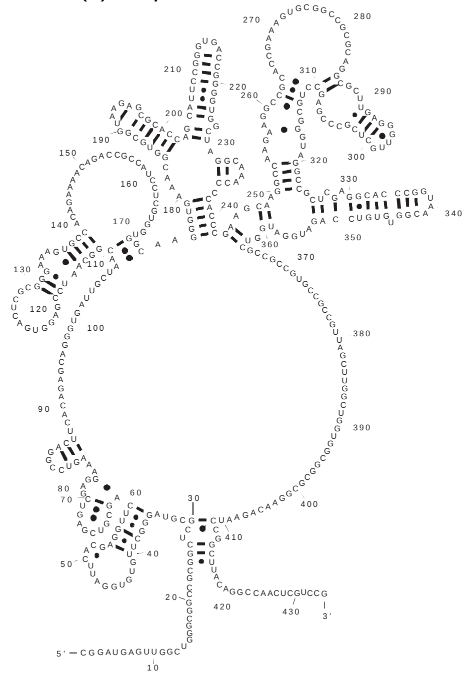

(d) *Mycobacterium tuberculosis*

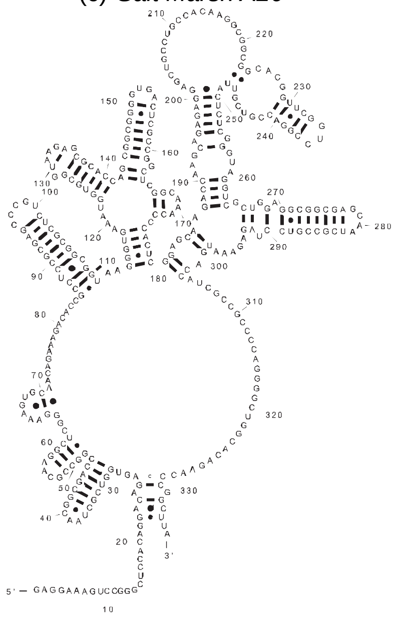

(e) *Lake Griffy A #8*

Figure S15. **Predicted structures by LocARNA for *Nocardioides albus*, *Propioniferax innocua*, *Salt Marsh A26*, *Mycobacterium tuberculosis* and *Lake Griffy A #8*.** Predicted structures for

Nocardioides albus (a), *Propioniferax innocua* (b), *Salt Marsh A26* (c), *Mycobacterium tuberculosis* (d) and *Lake Griffy A #8* (e). 5’ and 3’ ends of sequences are indicated by “5’” and “3’”. A heavy line between two nucleotides indicates the base pairing interaction between the nucleotides.

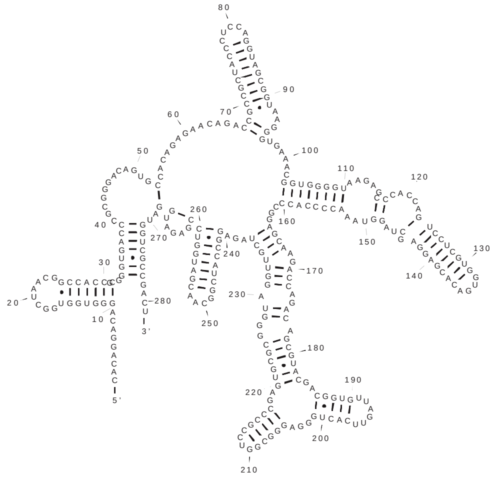

(a) *Nocardioides albus*

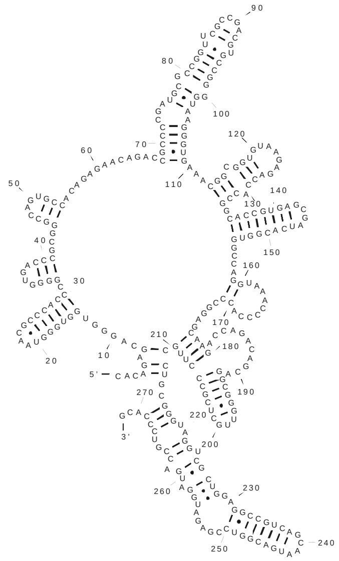

(b) *Propioniferax innocua*

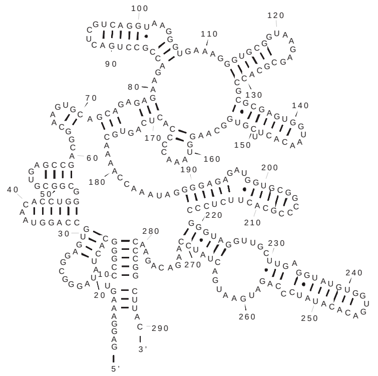

(c) *Salt Marsh A26*

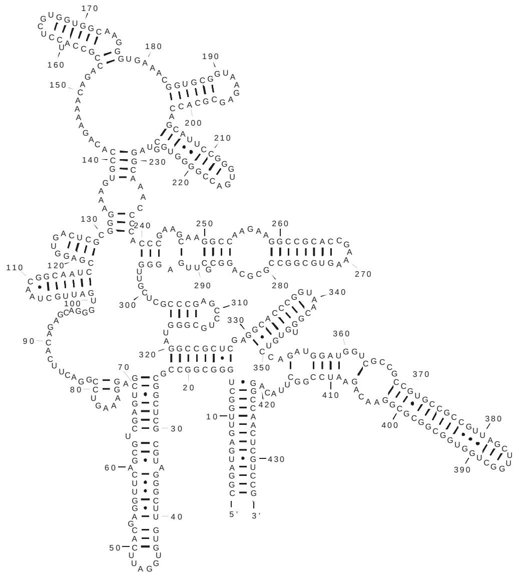

(d) *Mycobacterium tuberculosis*

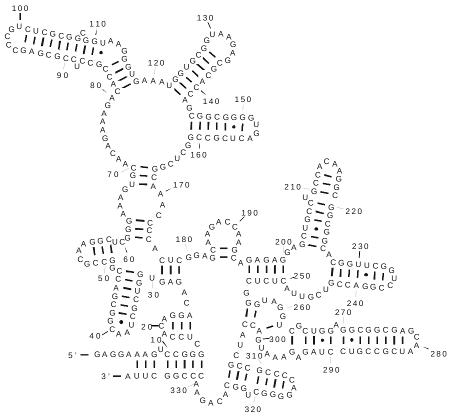

(e) *Lake Griffy A #8*

Figure S16. **Predicted structures by MaxExpect for *Nocardioides albus*, *Propioniferax innocua*, *Salt Marsh A26*, *Mycobacterium tuberculosis* and *Lake Griffy A #8*.** Predicted structures for

Nocardioides albus (a), *Propioniferax innocua* (b), *Salt Marsh A26* (c), *Mycobacterium tuberculosis* (d) and *Lake Griffy A #8* (e). 5’ and 3’ ends of sequences are indicated by “5’” and “3’”. A heavy line between two nucleotides indicates the base pairing interaction between the nucleotides.

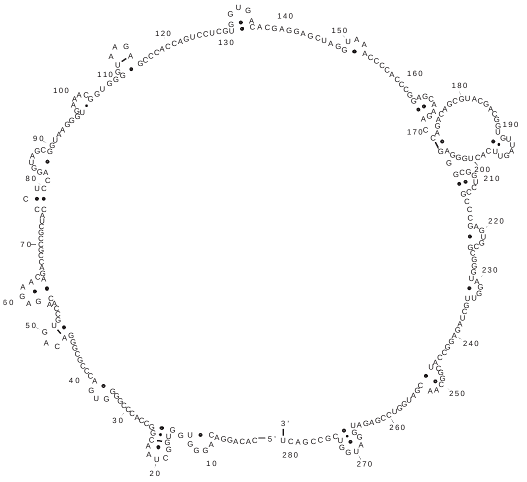

(a) *Nocardioides albus*

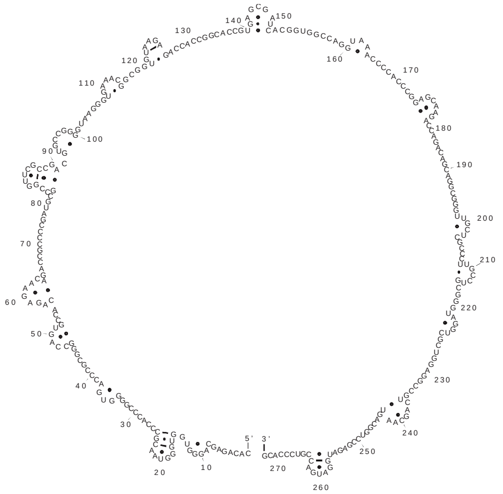

(b) *Propioniferax innocua*

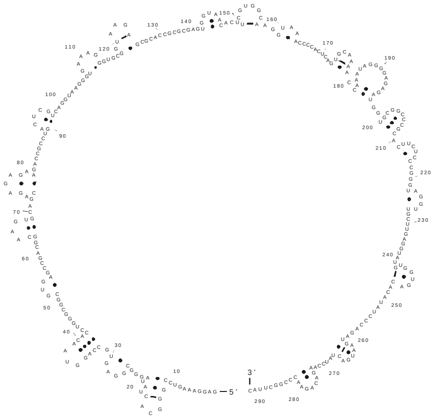

(c) *Salt Marsh A26*

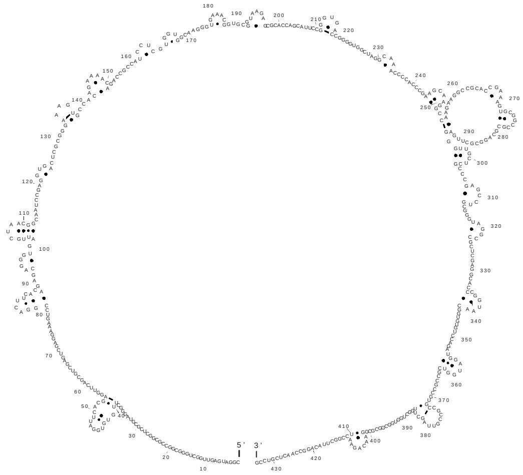

(d) *Mycobacterium tuberculosis*

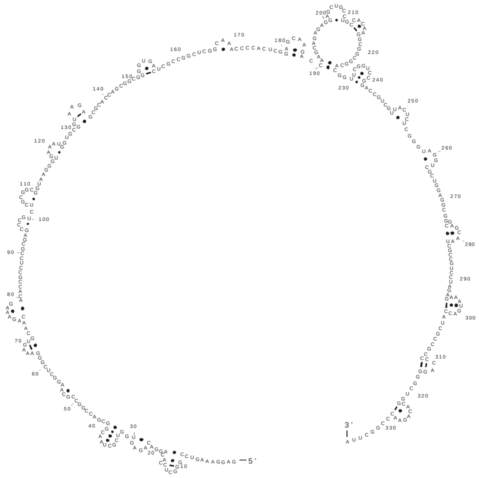

(e) *Lake Griffy A #8*

Figure S17. **Predicted structures by MXSCARNA for *Nocardioides albus*, *Propioniferax innocua*, *Salt Marsh A26*, *Mycobacterium tuberculosis* and *Lake Griffy A #8*.** Predicted structures for *Nocardioides albus* (a), *Propioniferax innocua* (b), *Salt Marsh A26* (c), *Mycobacterium*

tuberculosis (d) and *Lake Griffy A #8* (e). 5’ and 3’ ends of sequences are indicated by “5’” and “3’”. A heavy line between two nucleotides indicates the base pairing interaction between the nucleotides.

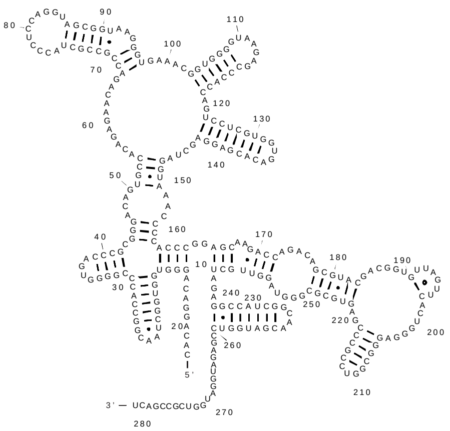

(a) *Nocardioides albus*

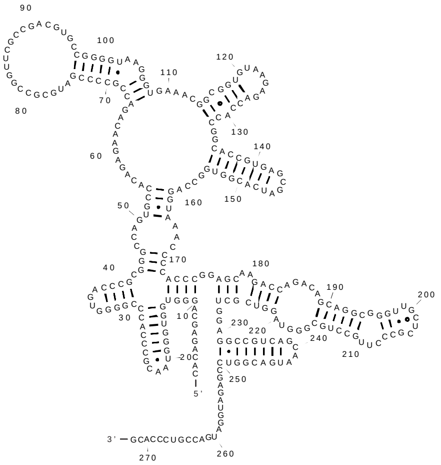

(b) *Propioniferax innocua*

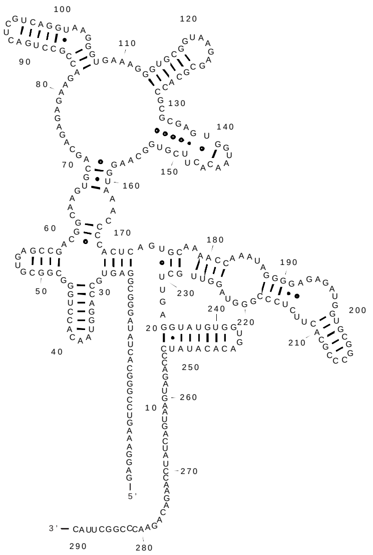

(c) *Salt Marsh A26*

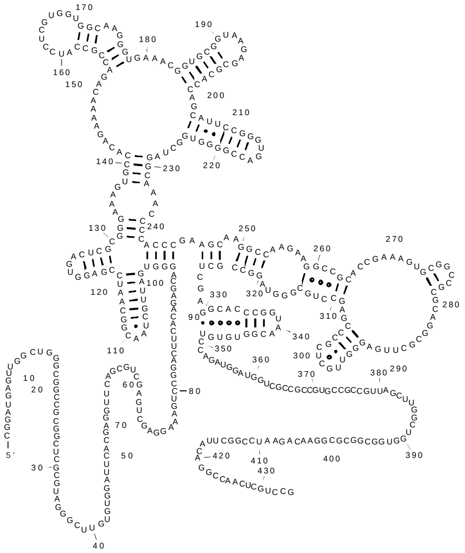

(d) *Mycobacterium tuberculosis*

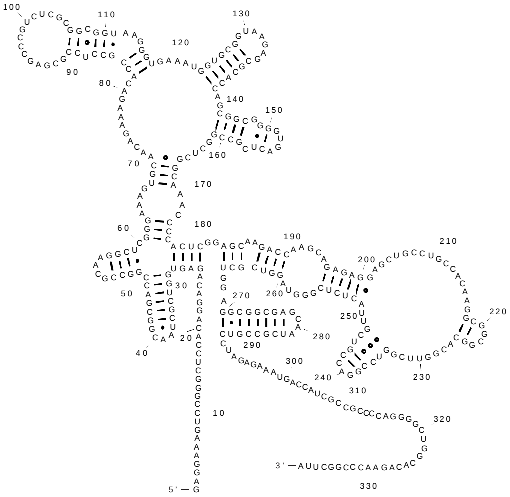

(e) *Lake Griffy A #8*

Figure S18. **Predicted structures by RNAalifold (using Clustal Omega) for *Nocardioides albus*, *Propioniferax innocua*, *Salt Marsh A26*, *Mycobacterium tuberculosis* and *Lake Griffy A #8*.**

Predicted structures for *Nocardioides albus* (a), *Propioniferax innocua* (b), *Salt Marsh A26* (c), *Mycobacterium tuberculosis* (d) and *Lake Griffy A #8* (e). 5’ and 3’ ends of sequences are indicated by “5’” and “3’”. A heavy line between two nucleotides indicates the base pairing interaction between the nucleotides.

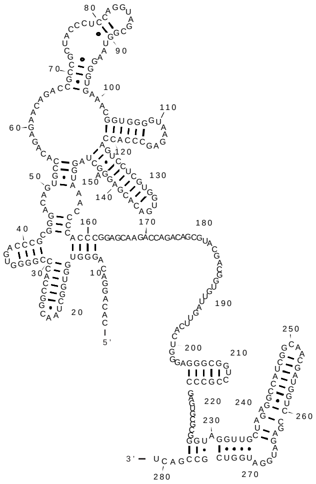

(a) *Nocardioides albus*

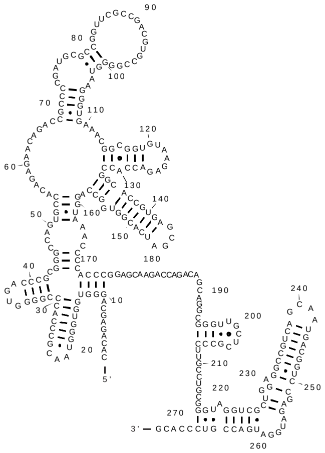

(b) *Propioniferax innocua*

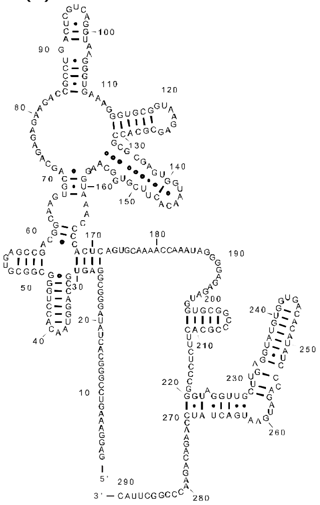

(c) *Salt Marsh A26*

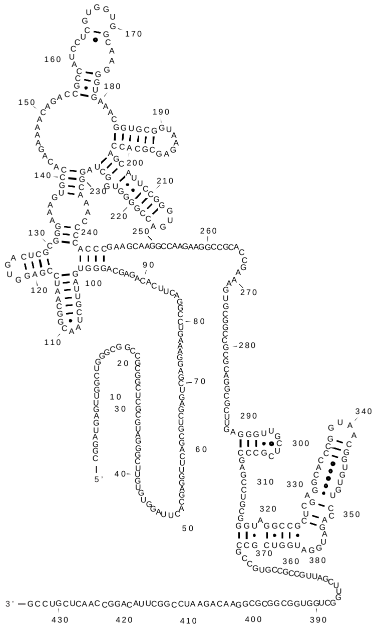

(d) *Mycobacterium tuberculosis*

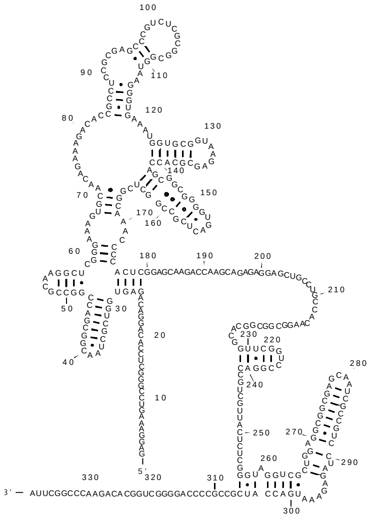

(e) *Lake Griffy A #8*

Figure S19. **Predicted structures by RNAalifold (using ClustalW) for *Nocardioides albus*, *Propioniferax innocua*, *Salt Marsh A26*, *Mycobacterium tuberculosis* and *Lake Griffy A #8*.**

Predicted structures for *Nocardioides albus* (a), *Propioniferax innocua* (b), *Salt Marsh A26* (c), *Mycobacterium tuberculosis* (d) and *Lake Griffy A #8* (e). 5’ and 3’ ends of sequences are indicated by “5’” and “3’”. A heavy line between two nucleotides indicates the base pairing interaction between the nucleotides.

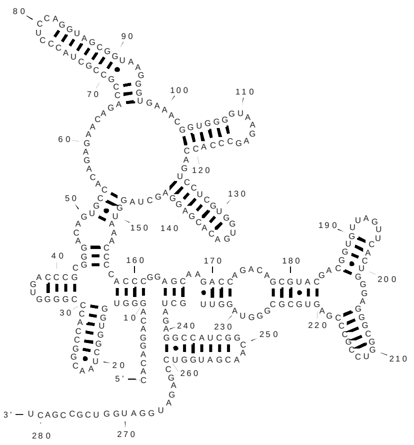

(a) *Nocardioides albus*

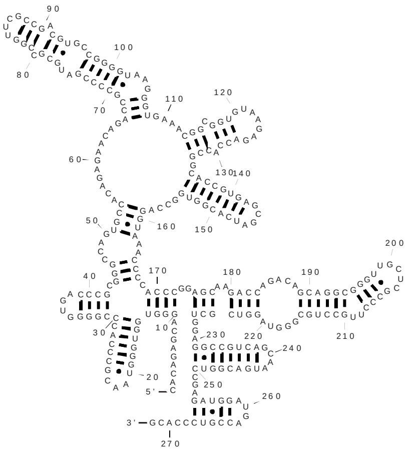

(b) *Propioniferax innocua*

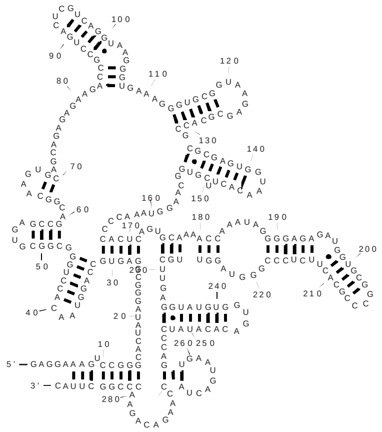

(c) *Salt Marsh A26*

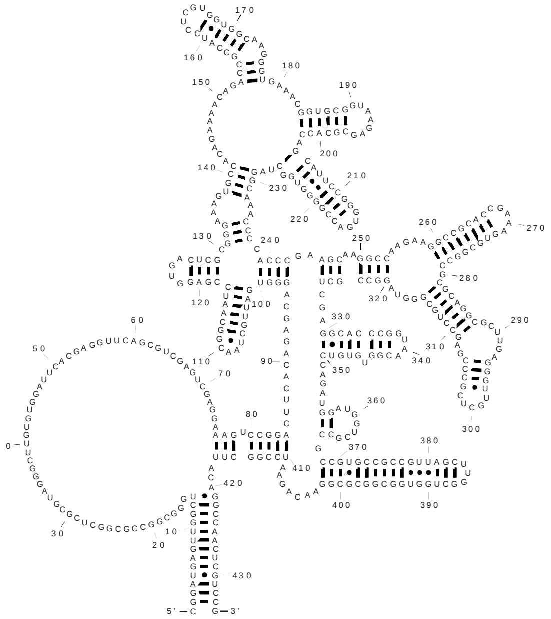

(d) *Mycobacterium tuberculosis*

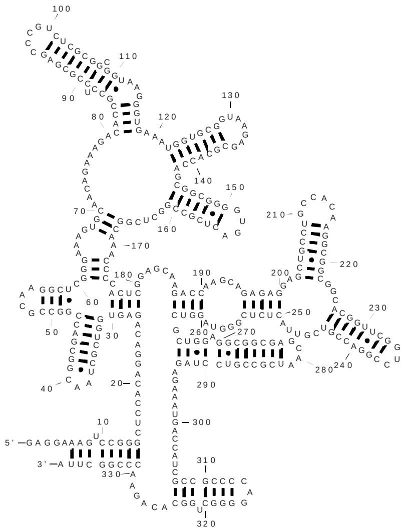

(e) *Lake Griffy A #8*

Figure S20. **Predicted structures by TurboFold for *Nocardioides albus*, *Propioniferax innocua*, *Salt Marsh A26*, *Mycobacterium tuberculosis* and *Lake Griffy A #8*.** Predicted structures for *Nocardioides albus* (a), *Propioniferax innocua* (b), *Salt Marsh A26* (c), *Mycobacterium*

tuberculosis (d) and *Lake Griffy A #8* (e). 5’ and 3’ ends of sequences are indicated by “5’” and “3’”. A heavy line between two nucleotides indicates the base pairing interaction between the nucleotides.

Section 7. Run time of TurboFold II

Table S3. Run time of the program over the selected families and different number of input sequences. The calculations were run on one core on a machine with an Intel® Core™ i7-4790 CPU @ 3.60GHz.

Family	*H=5 sequences*	*H=10 sequences*	*H=20 sequences*
tRNA	2.57s	7.31s	23.8s
RNase P	55.89s	2m3.67s	9m52.87s
Small subunit RNA	51m3.04s	1h52m30s	7h14m46s

References:

1. Harmanci, A.O., Sharma, G. and Mathews, D.H. (2007) Efficient pairwise RNA structure prediction using probabilistic alignment constraints in Dynalign. *BMC Bioinformatics*, **8**, 130.
2. Durbin, R., Eddy, S.R., Krogh, A. and Mitchison, G. (1998) *Biological sequence analysis: probabilistic models of proteins and nucleic acids*. Cambridge University Press, Cambridge, United Kingdom.
3. Gutell, R.R. (1994) Collection of small subunit (16S- and 16S-like) ribosomal RNA structures: 1994. *Nucleic Acids Res.*, **22**, 3502-3507.
4. Szymanski, M., Barciszewska, M.Z., Erdmann, V.A. and Barciszewski, J. (2002) 5S Ribosomal RNA Database. *Nucleic Acids Res.*, **30**, 176-178.
5. Zhou, Y., Lu, C., Wu, Q.J., Wang, Y., Sun, Z.T., Deng, J.C. and Zhang, Y. (2008) GISSD: Group I Intron Sequence and Structure Database. *Nucleic Acids Res.*, **36**, D31-37.
6. Brown, J.W. (1999) The Ribonuclease P Database. *Nucleic Acids Res.*, **27**, 314.
7. Rosenblad, M.A., Gorodkin, J., Knudsen, B., Zwieb, C. and Samuelsson, T. (2003) SRPDB: Signal Recognition Particle Database. *Nucleic Acids Res.*, **31**, 363-364.
8. Nawrocki, E.P., Burge, S.W., Bateman, A., Daub, J., Eberhardt, R.Y., Eddy, S.R., Floden, E.W., Gardner, P.P., Jones, T.A., Tate, J. *et al.* (2015) Rfam 12.0: updates to the RNA families database. *Nucleic Acids Res.*, **43**, D130-137.
9. Zwieb, C., Gorodkin, J., Knudsen, B., Burks, J. and Wower, J. (2003) tmRDB (tmRNA database). *Nucleic Acids Res.*, **31**, 446-447.
10. Juhling, F., Morl, M., Hartmann, R.K., Sprinzl, M., Stadler, P.F. and Putz, J. (2009) tRNAdb 2009: compilation of tRNA sequences and tRNA genes. *Nucleic Acids Res.*, **37**, D159-162.
11. Gardner, P.P., Wilm, A. and Washietl, S. (2005) A benchmark of multiple sequence alignment programs upon structural RNAs. *Nucleic Acids Res.*, **33**, 2433-2439.
